# Arrhythmia initiation in catecholaminergic polymorphic ventricular tachycardia type 1 depends on both heart rate and sympathetic stimulation

**DOI:** 10.1371/journal.pone.0207100

**Published:** 2018-11-06

**Authors:** Tore K. Danielsen, Ravinea Manotheepan, Mani Sadredini, Ida S. Leren, Andrew G. Edwards, Kevin P. Vincent, Stephan E. Lehnart, Ole M. Sejersted, Ivar Sjaastad, Kristina H. Haugaa, Mathis K. Stokke

**Affiliations:** 1 Institute for Experimental Medical Research, Oslo University Hospital and University of Oslo, Oslo, Norway; 2 K.G. Jebsen Center for Cardiac Research, University of Oslo, Oslo, Norway; 3 Center for Cardiological Innovation, Department of Cardiology, Oslo University Hospital, Rikshospitalet, Oslo, Norway and University of Oslo, Oslo, Norway; 4 Simula Research Laboratory, Oslo, Norway; 5 Heart Research Center Göttingen, Department of Cardiology and Pulmonology, University Medical Center Göttingen, Göttingen, Germany; 6 DZHK (German Centre for Cardiovascular Research), partner site Göttingen, Göttingen, Germany; 7 Institute for Surgical Research, Oslo University Hospital, Rikshospitalet, University of Oslo, Oslo, Norway; University of Canberra, AUSTRALIA

## Abstract

**Aims:**

Catecholaminergic polymorphic ventricular tachycardia type 1 (CPVT1) predisposes to ventricular tachyarrhythmias (VTs) during high heart rates due to physical or psychological stress. The essential role of catecholaminergic effects on ventricular cardiomyocytes in this situation is well documented, but the importance of heart rate *per se* for arrhythmia initiation in CPVT1 is largely unexplored.

**Methods and results:**

Sixteen CPVT1 patients performed a bicycle stress-test. Occurrence of VT triggers, i.e. premature ventricular complexes (PVC), depended on high heart rate, with individual thresholds. Atrial pacing above the individual PVC threshold in three patients did not induce PVCs.

The underlying mechanism for the clinical observation was explored using cardiomyocytes from mice with the *RyR2*-R2474S (RyR2-RS) mutation, which exhibit exercise-induced VTs. While rapid pacing increased the number of Ca^2+^ waves in both RyR2-RS and wild-type (p<0.05), β-adrenoceptor (βAR) stimulation induced more Ca^2+^ waves in RyR2-RS (p<0.05). Notably, Ca^2+^ waves occurred despite decreased sarcoplasmic reticulum (SR) Ca^2+^ content in RyR2-RS (p<0.05), suggesting increased cytosolic RyR2 Ca^2+^ sensitivity.

A computational model of mouse ventricular cardiomyocyte electrophysiology reproduced the cellular CPVT1 phenotype when RyR2 Ca^2+^ sensitivity was increased. Importantly, diastolic fluctuations in phosphorylation of RyR2 and SR Ca^2+^ content determined Ca^2+^ wave initiation. These factors were modulated towards increased propensity for arrhythmia initiation by increased pacing rates, but even more by βAR stimulation.

**Conclusion:**

In CPVT1, VT propensity depends on individual heart rate thresholds for PVCs. Through converging data from clinical exercise stress-testing, cellular studies and computational modelling, we confirm the heart rate-independent pro-arrhythmic effects of βAR stimulation in CPVT1, but also identify an independent and synergistic contribution from effects of high heart rate.

## 1. Introduction

Patients with catecholaminergic polymorphic ventricular tachycardia (CPVT) have an increased risk of sudden cardiac death due to ventricular arrhythmias. The mortality rate in untreated patients is 30–33% by the age of 35.[1] Current treatment options comprise β-adrenoceptor (βAR) antagonists and flecainide.[2–4] In earlier studies, as many as 46% of patients treated with βAR antagonists experienced breakthrough ventricular tachycardias (VT).[5] Flecainide offers effective added protection, but some patients still experience breakthrough VTs even on combined treatment.[2, 6, 7] In such patients, or patients who do not tolerate treatment with βAR antagonists, left cardiac sympathetic denervation can be an option.[8–10] If serious arrhythmic events occur despite optimal medical treatment, an implantable cardioverter defibrillator (ICD) is recommended,[8] but involves a risk of inappropriate shocks that can lead to patient distress and initiate VT and death.[3] Thus, new therapeutic strategies are needed, based on improved mechanistic insight.

Stress testing of patients with CPVT, a central diagnostic strategy, shows a relationship between increasing heart rate and the occurrence of ventricular ectopy.[1, 5] More severe arrhythmias are often observed during high heart rates, such as sustained VTs appearing above a certain heart rate threshold.[1] βAR stimulation has been identified as an important factor for the development of arrhythmias in CPVT, and catecholamines, i.e. adrenaline [11] or isoprenaline (ISO), infusion [12] has been used as a stress test in CPVT. However, the diagnostic polymorphic non-sustained VT was only induced in 31% of patients with mutations pathogenic for CPVT.[5] *In vivo*, βAR stimulation increases heart rate,[13] but also has important non-chronotropic, i.e. heart-rate independent, effects on ventricular cardiomyocytes. On the other hand, increased heart rate has important effects on ventricular cardiomyocytes that are independent of βAR stimulation. Therefore, clarifying the relative importance of the heart rate and sympathetic activity for development of arrhythmias in CPVT could have important implications for diagnostic procedures and treatment strategies.

The focus of this study is CPVT type 1 (CPVT1), caused by mutations in the gene encoding the major intracellular cardiac Ca^2+^ release channel, i.e. the ryanodine receptor 2 (RyR2).[14] *RyR2* mutations in patients with CPVT1 cause pathological Ca^2+^ leak from the sarcoplasmic reticulum (SR) in ventricular cardiomyocytes.[15, 16] Diastolic SR Ca^2+^ leak may lead to delayed afterdepolarization (DAD) and trigger ventricular arrhythmias.[15] Theoretically, βAR stimulation and high heart rate can increase the amplitude of DADs, and promote triggered activity.[17] Accumulating evidence indicates that Ca^2+^/calmodulin-dependent protein kinase II (CaMKII) could be a common mediator for the effects of both heart rate and βAR stimulation.[18, 19] CaMKII-dependent phosphorylation increases RyR2 channel opening probability, and thus the propensity for increased SR Ca^2+^ leak and arrhythmogenic Ca^2+^ waves.[20] Indeed, inhibition of CaMKII has proved beneficial in models of CPVT1.[19]

We hypothesized that both heart rate and βAR stimulation contribute independently to the development of ventricular arrhythmias in CPVT1. We tested this hypothesis by combining observations from patients, cellular experiments and mathematical modeling.

## 2. Methods

### 2.1 Patients and patient data

Patients with genetically confirmed CPVT1 were included through the Department of Cardiology, Oslo University Hospital Rikshospitalet. The study was approved by the Regional Committee for Medical and Health Research Ethics (REC-South-East; REC ID 201772 / 2011–19297), and conformed to the declaration of Helsinki. Written informed consent was obtained from all enrolled patients.

Sixteen patients performed standardized bicycle stress testing using a protocol previously described.[21, 22] Briefly, 12-lead ECGs were recorded during bicycling with increasing workload (Schiller CS-200 Ergo-Spiro, Diacor), starting at 25 W with stepwise increase until exhaustion. One to four tests per patient were included in the study. The threshold heart rate for ventricular arrhythmias in individual patients was defined as the heart rate at which premature ventricular complexes (PVC) occurred as bigeminy, couplets, or VT during stress testing. If patients did not develop any of these arrhythmic events, the threshold was set as the heart rate were single PVCs occurred.

Three patients with ICDs volunteered for an ICD-based pacing protocol following the bicycle stress test. In accordance with approval from the regional Ethical Committee, the pacing procedure was performed as part of the standard follow-up of these patients, and with a minimum of intervention. We wanted to assess the heart rate for start of ventricular arrhythmias before the pacing, to be able to choose the correct rate. Therefore, the exercise stress test had to be performed first and according to standard follow-up protocol. After cessation of the exercise test patients rested in the supine position until recovery of baseline heart rate, and for at least 10 minutes before the pacing procedure was performed as an add-on to their standard ICD control. Electrical pacing through the atrial electrode was performed for 30 s at 5–10 beats per minute (b.p.m.) above the individual threshold heart rate for ventricular arrhythmias identified during the bicycle stress test. A 12-lead ECG was recorded continuously during the ICD-pacing protocol.

### 2.2 Animal model of CPVT1

This project was approved by the Norwegian National Committee for Animal Welfare under the Norwegian Animal Welfare Act (FOTS ID: 7169, 5669), and conformed to the National Institute of Health guidelines (NIH publication No. 85–23, revised 1996, US). The generation of knock-in mice with a human CPVT1 causative *RyR2*-R2474S (RyR2-RS) mutation used in this study has been described previously.[16]

### 2.3 Cellular experiments

Mice were anaesthetized in 2% isoflurane inhalation prior to sacrifice by cervical dislocation. Left ventricular cardiomyocytes were isolated using constant flow perfusion of the coronary arteries with collagenase-containing solution as previously described.[23]

Whole-cell Ca^2+^ imaging in field-stimulated cardiomyocytes was performed with a PTI Microscope Photometer D-104G (PhotoMed, Køge, Denmark). Confocal line-scan images were recorded using a Zeiss *LSM 7* Live confocal microscope (Zeiss Observer Z1, Micro imaging, GmbH, Germany). All experiments were performed at 37°C and the experimental superfusate was based on a modified Hepes-Tyrode`s solution containing (in mM): Hepes 5, NaCl 140, KCl 5.4, MgCl_2_ 0.5, Glucose 5.5, NaH_2_PO_4_ 0.4, CaCl_2_ 1. pH was adjusted to 7.4 with NaOH. Fluo-4AM (5 μM, Molecular Probes, Eugene, Oregon, USA) was used to visualize cytosolic Ca^2+^, and isoprenaline (200 nM, NAF, Norway) for βAR stimulation.

### 2.4 Western blots

Hearts used for protein analysis were mounted on a modified Langendorff setup, and perfused through the aorta with a 37°C modified Hepes-Tyrode`s solution. The hearts were then paced at 4 or 8 Hz for three min, i.e. the same duration as the protocol for cellular experiments. This frequency was based on pilot experiments, and chosen to allow stable pacing. The frequency of activation was confirmed by simultaneous ECG recordings by electrodes from telemetric ECG transmitters (Data Sciences International, St. Paul, USA). After three min of pacing, the left ventricle was isolated, rapidly frozen in liquid nitrogen, and stored at –80°C. For βAR stimulation, hearts were perfused with ISO (200 nM) for 1 min.

Western blotting was performed with total protein homogenates from left ventricles, as previously described.[23]

### 2.5 Computer model

To quantitatively explore the effects of heart rate and βAR stimulation on Ca^2+^ handling in ventricular myocytes, we employed a computational model of mouse ventricular myocyte electrophysiology previously published by Morotti *et al*.[24] This model includes detailed representations of all membrane ion channels, as well as phospholemman, RyR2, the sarcoplasmic reticulum Ca^2+^ ATPase, phospholamban (PLB) and Troponin I. Importantly, this model also includes detailed and dynamic representations of protein kinase A (PKA) and CaMKII activity and their regulation of these ion channels and Ca^2+^ handling proteins. To model RyR2-RS cardiomyocytes, the Morotti computational model was only altered by increasing RyR2 luminal Ca^2+^ sensitivity until the model reproduced Ca^2+^ wave frequency and latency measured in RyR2-RS during cellular experiments.

Briefly, the Morotti RyR2 formulation is an extension of the 4-state model of Shannon *et al*.[25] for which RyR2 SR luminal Ca^2+^ sensitivity is calculated as a sigmoidal function of the luminal Ca^2+^ concentration. This sensitivity can be modulated by the half maximal effective concentration (EC50) for luminal Ca^2+^, which increases the RyR2 closed-to-open transition rate, and reciprocally reduces the open-to-inactive transition rate. The EC50 was the only parameter we modulated to fit experimental RyR2-RS data. We simulated a range of increased Ca^2+^ sensitivities, from 2–30% above the value in the original Morotti model, to identify the Ca^2+^ sensitivity that best fit the experimental data on the latency of Ca^2+^ wave after pacing and the number of Ca^2+^ waves in the post-pacing period, and remained in qualitative agreement with steady state Ca^2+^ handling. The simulations for RyR2-RS presented in this article were all run with a constant EC50 value, which was decreased by 10% compared to WT.

### 2.6 Statistics

Results are reported as mean ± standard error of mean (SEM). Ca^2+^ sparks data are reported by density plots using kernel density smoothing with a bandwidth of 0.3 in R software (version 3.0.2, The R Foundation for Statistical Computing). ANOVA or Nested ANOVA analysis with Bonferroni corrections were used as appropriate for analysis of RR-intervals in CPVT1 patients and cellular experiments, except analysis of Ca^2+^ spark frequency for which Poisson analysis was used to adjust for a skewed distribution. P<0.05 was considered statistically significant.

## 3. Results

### 3.1 Ventricular arrhythmias in patients with CPVT1 were associated with increased heart rates during bicycle testing, but not during direct pacing

Sixteen patients (39±4 y, 56% women) diagnosed with CPVT1 were included. All patients were positive for CPVT-associated RyR2 mutations, two were probands and 14 were identified as part of family screening. Of these, 11 patients (69%) had symptoms associated with arrhythmias. All patients had been evaluated according to guidelines with echocardiography, and seven patients (44%) also with cardiac MR as part of the initial evaluation.(Table 1).

**Table 1:** Patient overview.

Patient	Test number	Gender	Age at diagnosis	Family member vs. Proband	Mutation	Symptoms	Family history	Comorbidities	Imaging	Medication	ICD	Age at- stress test	No βAR antagonist		With βAR antagonist	
													Baseline	RR interval before first arrhythmic event	Baseline	RR interval before first arrhythmic event
1	I	Woman	43	Family member	G2337V 46 RyR2	Palpitations	SCD daughter 13y; SCD brother 21y	No	Normal echo	No	No	43	880	440		
	II					no				Metoprolol succinate 100 mg x 1	No	44			980	
2	I	Woman	38	Family member	G4671V 97 RyR2	Syncope while swimming	Son with cardiac symptoms	No	Normal echo; Normal CMR	No	No	38	940	340		
	II					No				Metoprolol succinate 100 mg x 1	No	39			820	390
3	I	Woman	29	Family member	G2337V 46 RyR2	Palpitations	SCD in family member with CPVT	No	Normal echo	No	No	28		360		
	II					No				Metoprolol succinate 100 mg x 1	No	29			1040	440
4	I	Woman	50	Family member	G2337V 46 RyR2	No	SCD son 24y	No	Normal echo; normal CMR	No	No	49				
	II					No				Metoprolol succinate 100 mg x 1	No	49				
	III					No				Metoprolol succinate 150 mg x 1	No	50				
5	I	Woman	58	Family member	G4671V 97 RyR2	Palpitations	Daughter and grandchild with CPVT	No	Mild mitral regurgitation on echo	No	No	56	980	440		
6	I	Man	56	Family member	G2337V 46 RyR2	No	SCD brother 10y; SCD brother 44y	No	Medium mitral regurgitation and mild aortic regurgitation on echo	Metoprolol succinate 100 mg x 1	No	55			1040	540
	II					No				Metoprolol succinate 150 mg x 1	No	56			1300	
	III					No				Metoprolol succinate 150 mg x 1	No	57			1320	560
7	I	Man	39	Family member	G2337V 46 RyR2	No	SCD several family members	No	Normal echo.; normal CMR	No	No	39	1040	420		
	II					No				Metoprolol succinate 50 mg x 1	No	40			940	430
	III					No				Carvedilol 25 mg x1	No	41			960	360
	IV					No				Carvedilol 25 mg x1	No	41			1180	400
8	I	Man	41	Family member	G2337V 46 RyR2	No	SCD several family members	No	Normal echo	No	No	41	840	460		
9	I	Man	36	Family member	G2337V 46 RyR2	Syncope during physical activity	SCD several family members	No	Normal echo	No	No	36	1200	440		
	II					No				Metoprolol succinate 100 mg x 1	No	36			1200	500
	III					No				Metoprolol succinate 100 mg x 1	No	37			1060	540
10	I	Woman	66	Family member	G2337V 46 RyR2	No	SCD grandchild with CPVT	Hypertention	Small aortic regurgitation on echo	Metoprolol succinate 50 mg x 2	No	68			1260	480
11	I	Man	22	Family member	G2337V 46 RyR2	No	SCD several family members	No	Normal echo	Metoprolol succinate 50 mg x 1	No	23	1100			
12	I	Man	19	Family member	G2337V 46 RyR2	No	SCD father and several family members	Allergies	Normal echo	No	No	19				
13	I	Man	35	Family member	G2337V 46 RyR2	Palpitations	SCD several family members	Hypothyroidism	Normal echo	Levothyroxine 50 mcg x 1	No	35	1260	440		
	II					No				Metoprolol succinate 100 mg x 1, Levothyroxin	No	35			1080	500
14	I	Woman	54	Family member	G2337V 46 RyR2	Palpitations	SCD in family member with CPVT	Hypothyroidism	Normal echo	Levothyroxine	No	54	1400			
	II					No				Metoprolol succinate 50 mg x 1, Levothyroxine	No	54			1960	640
	III					Palpitations				Metoprolol succinate 50 mg x 1, Levothyroxine	No	56			1760	
15	I	Man	13	Proband	G4671V 97 RyR2	Ventricular fibrillation during svimming		No	Small aortic regurgitation on echo.; Normal CMR	Metoprolol succinate 100 mg x 2	No	17	680			
16	I	Woman	18	Proband	R176Q 8 RyR2	Multiple syncopes	SCD brother	No	Normal echo	Nadolol 40 mg x 1	No	26			1200	560
**Average**													1032 ± 68	418 ± 15*	1194 ± 74	488 ± 22*
**Patients with ICD included in pacing protocol**																
17		Woman	39	Family member	G2337V 46 RyR2	Four episodes of syncope during swimming and physical activity	SCD son 10y	No	Normal echo.; normal CMR	Metoprolol succinate 50 mg x 1	Yes	48				
18		Man	13	Proband	F4176S 90 RyR2	Multiple syncopes		No	Normal echo	Nadolol 80 mg + 60 mg, Flecainide 100 mg x 2, Methylphenidate	Yes	18				
19		Man	15	Family member	R176Q 8 RyR2	Multiple syncopes	Sister with CPVT	No	Normal echo, normal CMR	Metoprolol succinate 75 mg x 2, Flecainide 100 mg x 2	Yes	20				

SCD, sudden cardiac death; CMR, cardiac magnetic resonance imaging.

*p<0.00001 vs. baseline.

**Fig 1:**
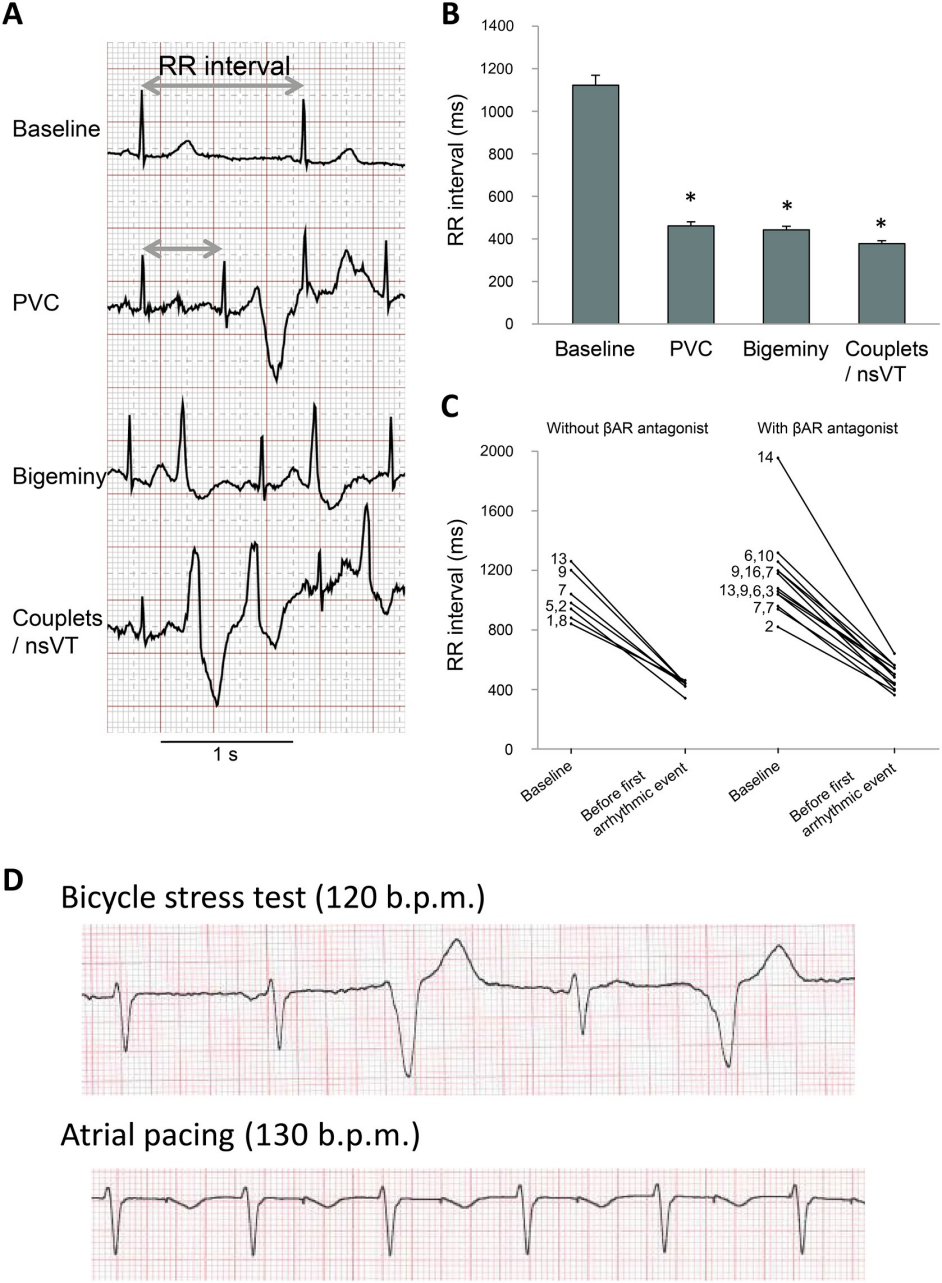
Bicycle stress testing showed the heart rate-dependence of arrhythmias in patients with CPVT1, while atrial pacing did not induce arrhythmias. (A) ECG tracings from a patient with CPVT1 recorded during a bicycle stress test. RR-interval was measured before PVCs occurred. (B) Bar graphs of RR-intervals of sinus beats in stable sinus rhythm (baseline), and immediately before the occurrence of isolated PVCs, PVCs in bigeminy, and couplets or nsVT, respectively. Data from 31 bicycle stress tests performed by 16 patients with CPVT1. *p<0.05 compared to baseline with one-way ANOVA with Bonferroni correction. (C) Individual plots of RR-intervals of patients who developed arrhythmias during the stress test. RR-intervals at baseline and before the first arrhythmic event are shown, both in absence and in presence of βAR antagonists. (D) ECG tracings from a patient with CPVT1 recorded during a bicycle stress test (upper panel) with PVCs in bigeminy at 120 beat per minute (b.p.m.), and at rest during pacing through an atrial lead with no PVCs at 130 b.p.m. (lower panel).

ECGs were recorded during bicycle stress tests from all patients. When available, we included results from multiple stress tests per patient. No patients exhibited PVCs or ventricular arrhythmias at rest, and the mean RR-interval at baseline was longer than the RR-interval immediately preceding any of the ventricular arrhythmias observed (p<0.05, Fig 1A–1C). This illustration of the heart rate dependence of ventricular arrhythmias in CPVT1 was seen both in untreated patients and in patients treated with a beta-adrenoceptor antagonist (Fig 1C).

In addition to the sixteen patients included based on available data from bicycle stress testing, we included three patients harboring an ICD implanted due to CPVT1 (Table 1). To test the importance of heart rate, we performed atrial pacing at rest in these patients. The pacing protocol was performed following bicycle testing after complete recovery to baseline heart rate. Atrial pacing was performed at 5–10 b.p.m. above the threshold for occurrence of PVCs during the bicycle stress test. None of the patients developed PVCs during pacing through the ICD (Fig 1D).

### 3.2 βAR stimulation revealed an increased propensity for arrhythmogenic Ca^2+^ release events in RyR2-RS mouse left ventricular cardiomyocytes

**Fig 2:**
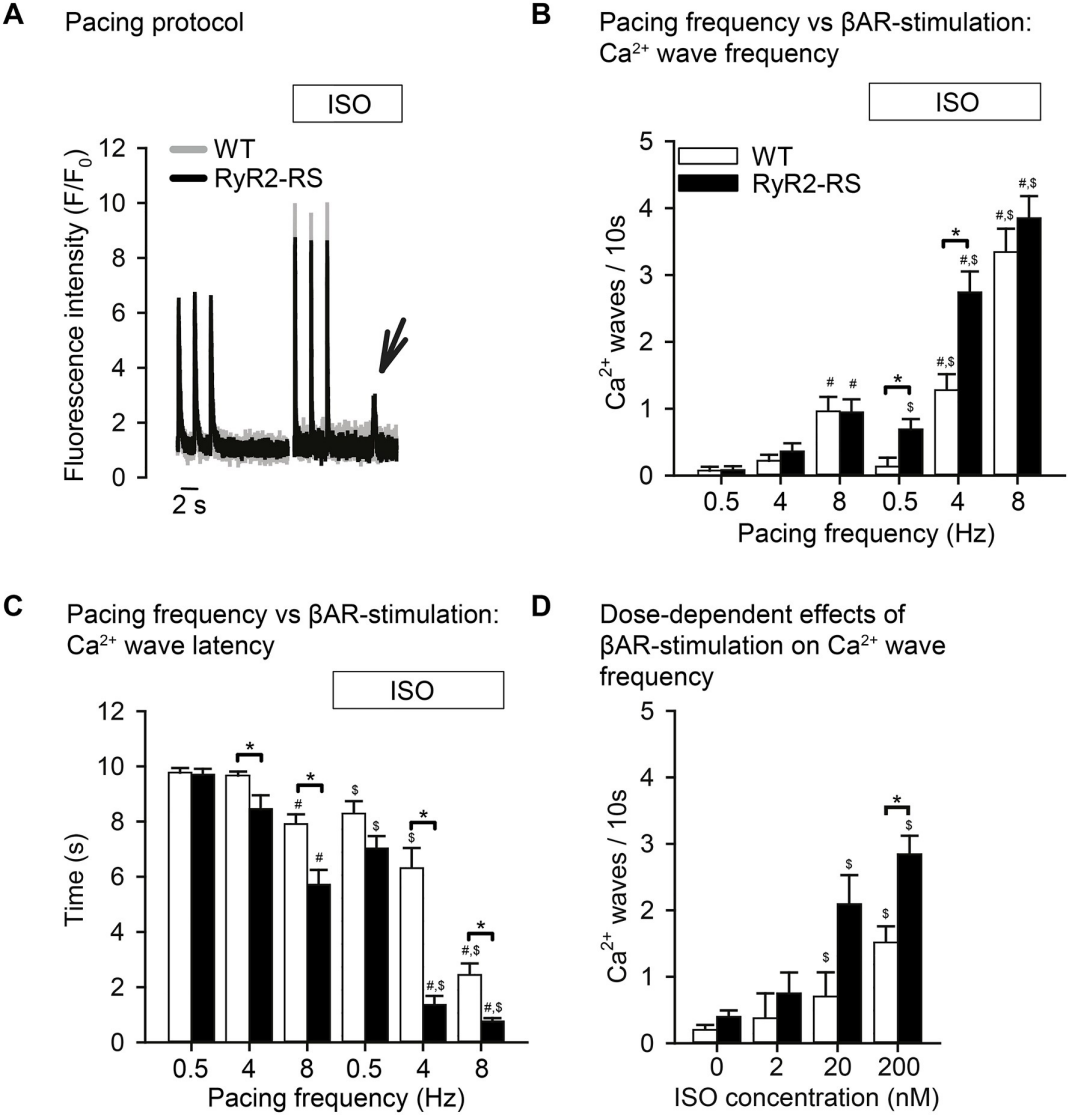
High pacing frequency induced Ca^2+^ waves in RyR2-RS mouse left ventricular cardiomyocytes, but βAR stimulation was necessary to reveal increased propensity compared to WT. (A) Tracings of whole-cell Ca^2+^ fluorescence showing Ca^2+^ transients and Ca^2+^ waves (arrow) during 0.5 Hz pacing by field stimulation in absence and presence of stimulation of βARs with ISO. (B) Bar graphs showing the mean frequency of Ca^2+^ waves in RyR2-RS and WT cardiomyocytes in a 10 s post-pacing period after 0.5, 4 and 8 Hz pacing in absence and presence of ISO, respectively. Analyzed by Nested ANOVA with data from 9 RyR2-RS and 8–15 WT mice per bar (29–55 cells per result). (C) Bar graphs showing the mean Ca^2+^ wave latency in RyR2-RS and WT cardiomyocytes after different pacing frequencies, in absence and presence of ISO. Analyzed by Nested ANOVA with data from 9–16 RyR2-RS and 10–21 WT mice per bar (28–70 cells per result). (D) Bar graph showing the mean frequency of Ca^2+^ waves in a 10 s period after 4 Hz pacing in 0, 2, 20 and 200 nM ISO. Analyzed by Nested ANOVA with data from 3–13 RyR2-RS and 3–18 WT mice (8–65 cells per result). *p<0.05 RyR2-RS vs WT, ^#^p<0.05 vs 0.5 Hz in the same conditions (+/- ISO), ^$^p<0.05 +ISO vs–ISO for the same genotype and frequency.

The propensity for diastolic Ca^2+^ waves in RyR2-RS and WT left ventricular cardiomyocytes was measured in a 10 s period after stable Ca^2+^ transients, which had been induced by field stimulation for 30 s (Fig 2A). In absence of βAR stimulation, progressive increase of the pacing frequency raised the number of Ca^2+^ waves in both RyR2-RS and WT (p<0.05), but did not reveal any differences between RyR2-RS and WT (Fig 2B). βAR stimulation, however, resulted in an increased number of Ca^2+^ waves in RyR2-RS compared to WT both at 0.5 and 4 Hz pacing (p<0.05, Fig 2B).

Another measurement of Ca^2+^ wave propensity is the time to occurrence of the first Ca^2+^ wave upon secession of pacing, i.e. post-pacing Ca^2+^ wave latency. This period decreased with pacing frequency in both RyR2-RS and WT (p<0.05), but was shorter in RyR2-RS than WT at 4 and 8 Hz both in the absence and presence of βAR stimulation (p<0.05, Fig 2C).

To test the dose-response relationship between βAR stimulation and the number of Ca^2+^ waves in the post-pacing period, cardiomyocytes were exposed to 0, 2, 20 and 200 nM ISO (4 Hz pacing, Fig 2D). Increasing ISO concentrations resulted in increased frequency of Ca^2+^ waves in both WT and RyR2-RS (p<0.05). Overall, the frequency of Ca^2+^ waves was higher in RyR2-RS than WT across ISO concentrations (p<0.05).

**Fig 3:**
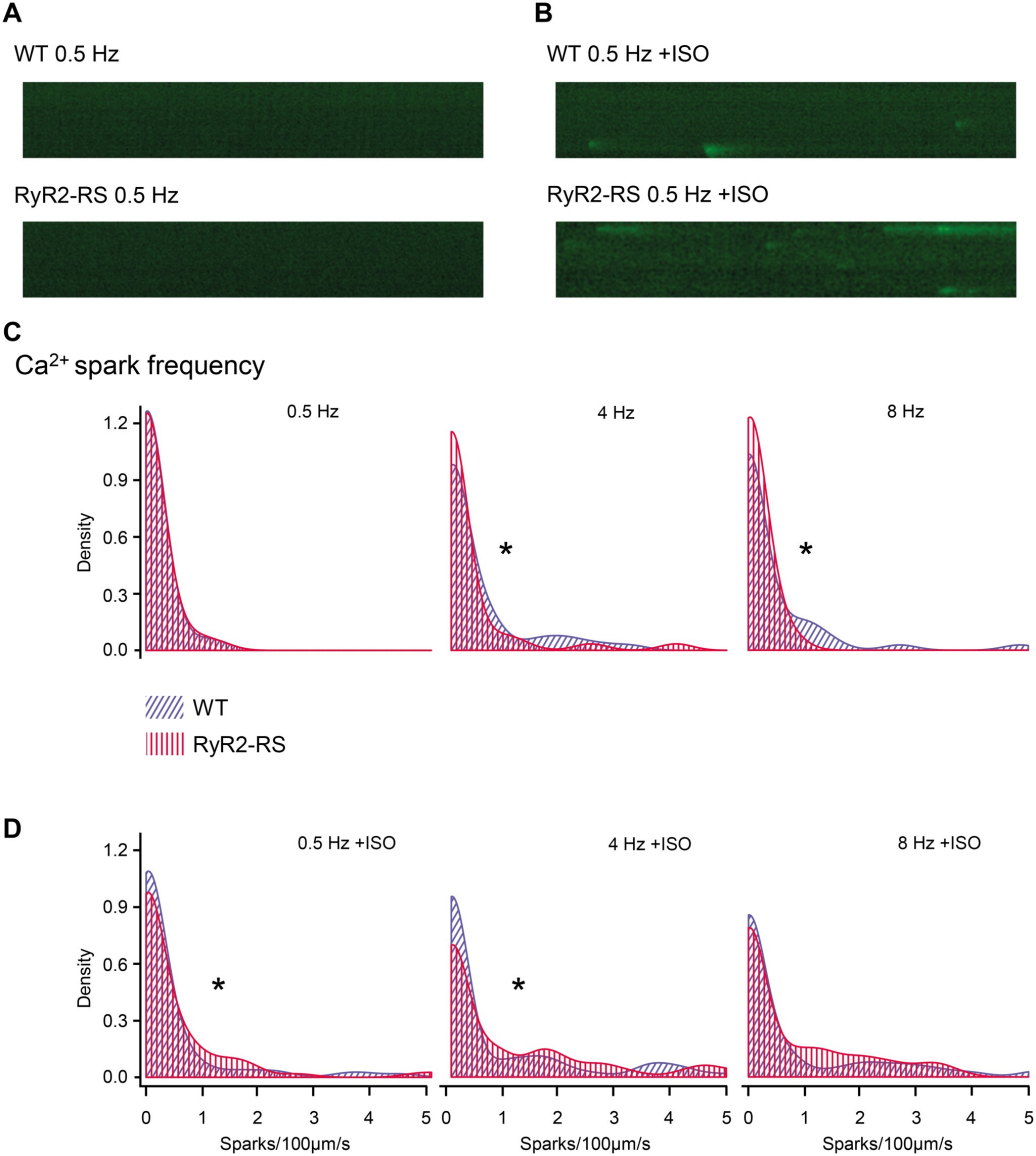
High pacing frequency induced Ca^2+^ sparks in RyR2-RS mouse left ventricular cardiomyocytes, but βARstimulation was necessary to reveal increased propensity compared to WT. (A) Line scan confocal imaging in absence of stimulation of βARs with ISO after 0.5 Hz pacing in WT (upper panel) and RyR2-RS (lower panel). (B) Line scan confocal imaging in presence of ISO after 0.5 Hz pacing in WT (upper panel) and RyR2-RS (lower panel). (C) and (D) Density plots illustrating the distribution of number of cells with 0–5 Ca^2+^ sparks per 100 μm per second, occurring after 0.5, 4 and 8 Hz pacing in absence and presence of ISO, respectively. Higher density means more cells. The legend shows how different patterns represent WT or RyR2-RS, respectively. Results from Poisson analysis of data from 13 RyR2-RS mice (47 cells) and 15 WT mice (62 cells), *p<0.05.

To further characterize diastolic SR Ca^2+^ release in RyR2-RS and WT, Ca^2+^ sparks were recorded by confocal microscopy (Fig 3A and 3B). In the absence of βAR stimulation, the propensity for Ca^2+^ sparks was low in both RyR2-RS and WT (Fig 3C), while during βAR stimulation the number of Ca^2+^ sparks increased in both groups and was higher in RyR2-RS compared to WT (p<0.05, Fig 3D).

### 3.3 SR Ca^2+^ content and threshold for Ca^2+^ waves were lower in RyR2-RS than WT

**Fig 4:**
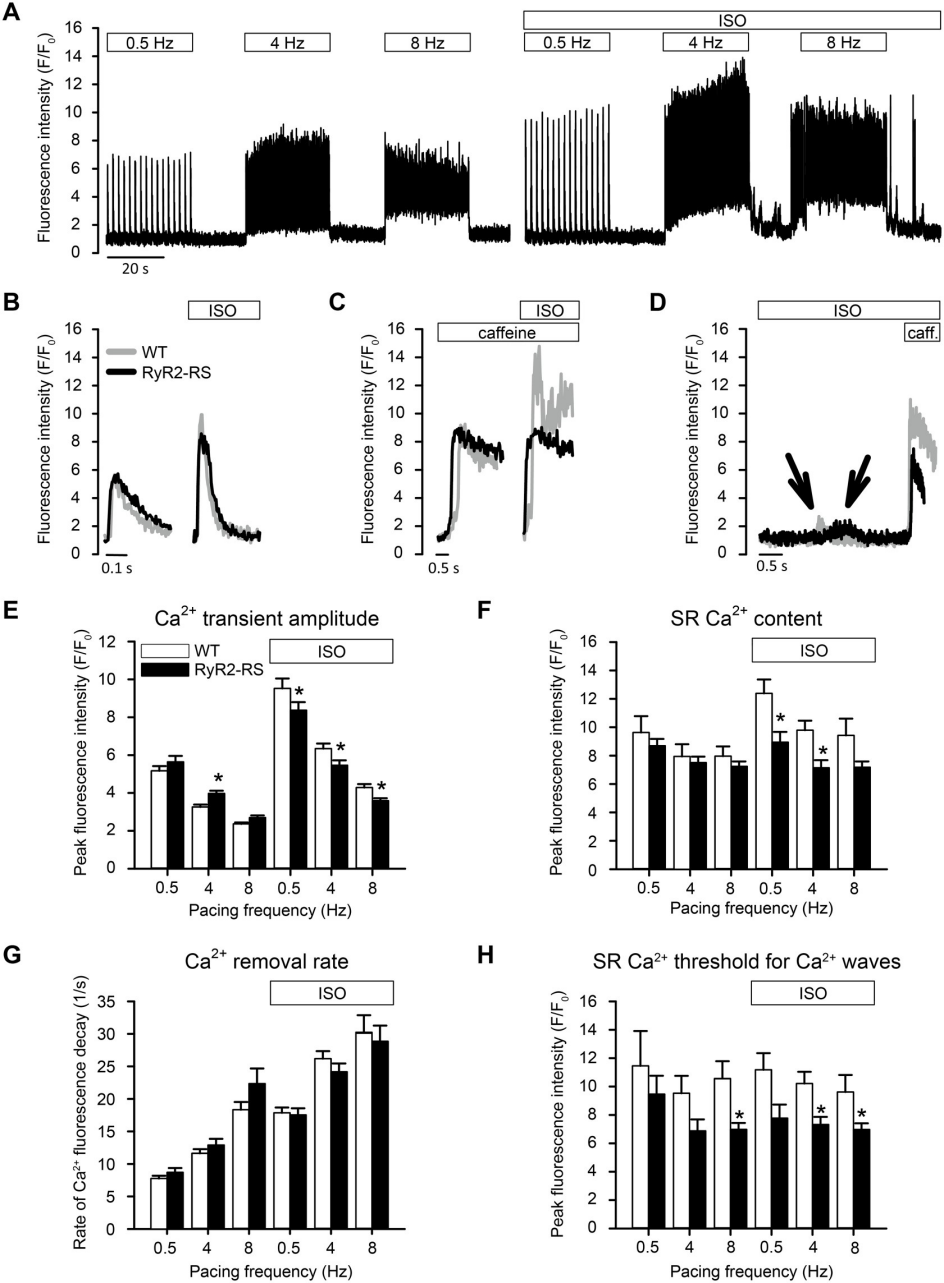
Ca^2+^ handling characteristics in RyR2-RS left ventricular cardiomyocytes indicated increased RyR open probability and lower threshold for diastolic Ca^2+^ release. (A) A whole-cell Ca^2+^ fluorescence tracing showing the experimental protocol with 0.5, 4 and 8 Hz pacing at baseline and during βAR stimulation. (B) Tracings of whole-cell Ca^2+^ fluorescence showing Ca^2+^ transients during 0.5 Hz pacing in absence and presence of ISO. (C) Tracings of whole-cell Ca^2+^ fluorescence showing caffeine-elicited Ca^2+^ release after 0.5 Hz pacing. Peak fluorescence intensity was used for measurements of SR Ca^2+^ content. Caffeine was added immediately after the last stimulated Ca^2+^ transient. (D) Tracings of whole-cell Ca^2+^ fluorescence showing caffeine-elicited Ca^2+^ release at the time of occurrence of Ca^2+^ waves after 4 Hz stimulation. Caffeine was added immediately after the occurrence of a Ca^2+^ wave. This protocol was used for measurements of threshold SR Ca^2+^ content. Peak fluorescence intensity was used for measurements of SR Ca^2+^ content. (E) Bar graphs showing mean Ca^2+^ transient amplitude at different pacing frequencies in absence and presence of ISO. Analyzed by Nested ANOVA with data from 8–10 RyR2-RS mice and 7–19 WT mice per bar (29–58 cells per result). (F) Bar graphs showing mean SR Ca^2+^ content at different pacing frequencies in absence and presence of ISO. Analyzed by Nested ANOVA with data from 7–10 RyR2-RS mice and 4–11 WT mice per bar (20–26 cells per result). (G) Bar graphs showing mean decay rates of the Ca^2+^ transients at different pacing frequencies in absence and presence of ISO. Analyzed by Nested ANOVA with data from 8–9 RyR2-RS mice and 7–19 WT mice per bar (29–58 cells per result). (H) Bar graphs showing mean threshold SR Ca^2+^ content at different pacing frequencies in absence and presence of ISO. Analyzed by Nested ANOVA with data from 6–7 RyR2-RS mice and 3–5 WT mice per bar (8–20 cells per result), except 0.5 Hz, at which a meaningful threshold was not obtained since very few cells exhibit Ca^2+^ waves. For this frequency, bar graphs represent data from 2 mice (3 cells) in each group. *p<0.05 RyR2-RS vs WT.

The effects of pacing and βAR stimulation on Ca^2+^ release and removal was further investigated by characterization of key aspects of Ca^2+^ handling in isolated left ventricular cardiomyocytes. This was studied by pacing and caffeine induced Ca^2+^ transients in RyR2-RS and WT (Fig 4A–4D). In absence of βAR stimulation, RyR2-RS developed higher Ca^2+^ transient amplitudes compared to WT at 4 Hz, while at 0.5 and 8 Hz, no significant differences were found (Fig 4E). Following βAR stimulation, however, the Ca^2+^ transient amplitude was lower in RyR2-RS compared to WT at all pacing frequencies (p<0.05, Fig 4E). In line with this, SR Ca^2+^ content in the absence of βAR stimulation was not significantly different in RyR2-RS and WT across pacing frequencies (Fig 4F), while following βAR stimulation, SR Ca^2+^ content was lower in RyR2-RS than WT overall (p<0.05), and at both 0.5 and 4 Hz (Fig 4F, p<0.05). These differences could not be explained by refilling of the SR, as cytosolic Ca^2+^ removal rate, a key determinant for the Ca^2+^ transient amplitude and SR Ca^2+^ content, was not significantly different between RyR2-RS and WT (Fig 4G).

Next, threshold SR Ca^2+^ content, defined as SR Ca^2+^ content at which Ca^2+^ waves occurred, was assessed (Fig 4D and 4H). Overall, Ca^2+^ waves developed at a lower SR Ca^2+^ content in RyR2-RS than in WT both in the absence (p<0.05) and presence of βAR stimulation (p<0.05).

### 3.4 Measurements of abundance of Ca^2+^ handling proteins did not reveal differences between RyR2-RS and WT

**Fig 5:**
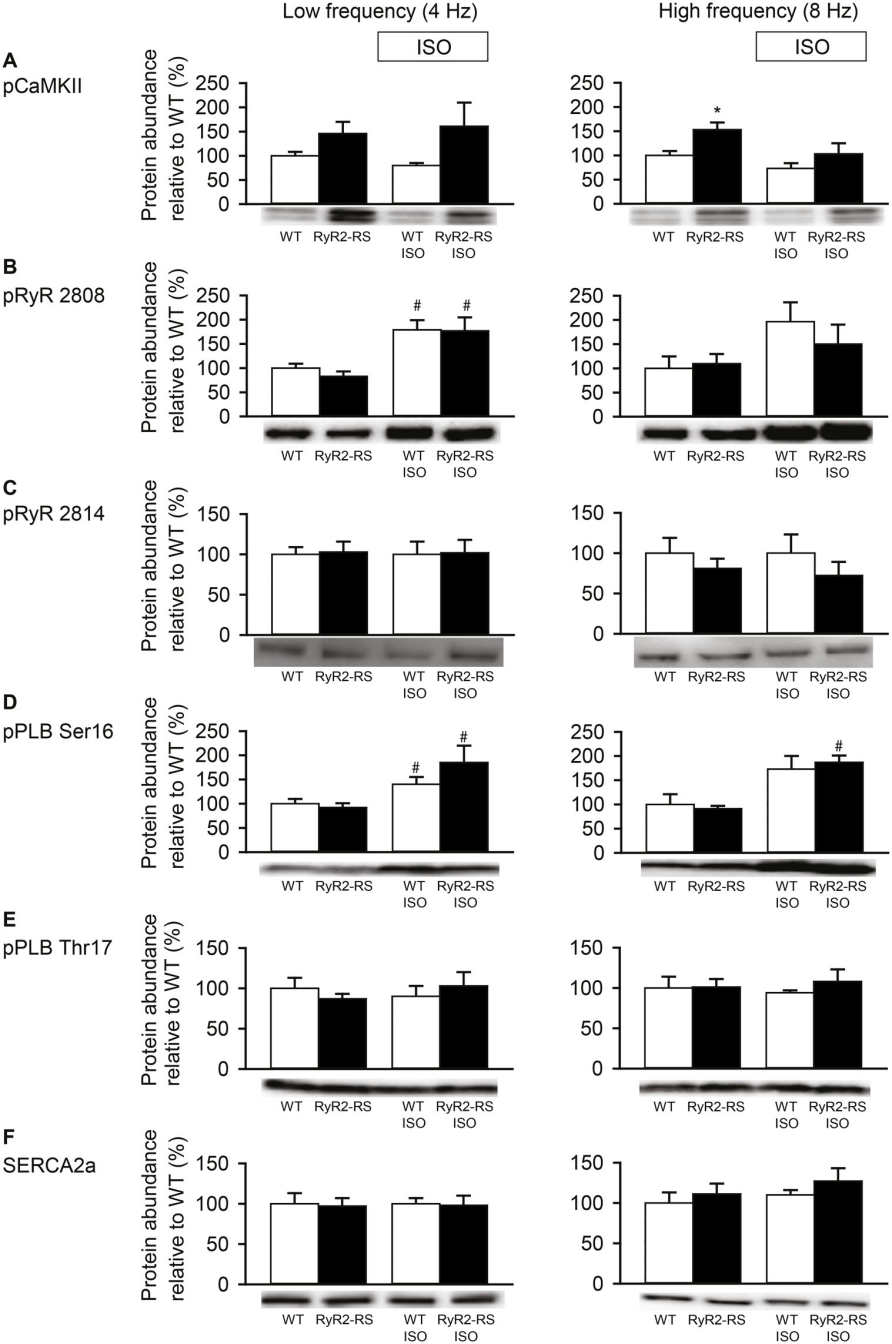
Analysis of key phosphoproteins did not show any differences between RyR2-RS and WT. Protein abundance was analyzed after 4 and 8 Hz pacing in absence and presence of ISO. (A) CaMKII phospho-threonine286 (pCaMKII), (B) RyR2 phospho-serine-2808 (pRyR2808), (C) RyR2 phospho-serine-2814 (pRyR 2814), (D) PLB phospho-serine 16 (pPLB-Ser16), (E) PLB phospho-threonine 17 (pPLB-Thr17), (F) SERCA2a. The graphs show mean protein abundance in 6 hearts from each group. Western blot results were normalized to WT (in the absence of ISO stimulation) at 4 and 8 Hz. *p<0.05, #p<0.05 vs. baseline in absence of ISO with Student`s T-test for unpaired data.

The abundance of key Ca^2+^ handling proteins and phosphoproteins were quantified in left ventricular tissue from Langendorff perfused and paced hearts (Fig 5 and S1 Fig). The only observed difference between the RyR-RS and WT was higher abundance of CaMKII phosphorylated at threonine286, i.e. autophosphorylated CaMKII, in RyR2-RS compared to WT at 8 Hz in the absence of βAR stimulation (p<0.05, Fig 5A). No significant differences between RyR2-RS and WT were observed with regard to the abundance of RyR2 phospho-serine2808, RyR2 phospho-serine2814, PLB phospho-serine16 or PLB phospho-threonine17 at 4 or 8 Hz pacing (Fig 5B–5E). SERCA2a abundance was also similar in RyR2-RS and WT (Fig 5F). Importantly for quality control, βAR stimulation increased PLB phospho-serine16 in both RyR2-RS and WT (p<0.05, Fig 5B and 5D).

### 3.5 Computer simulations support that pacing rate and βAR stimulation have independent effects on the propensity for Ca^2+^ waves in both RyR2-RS and WT

**Fig 6:**
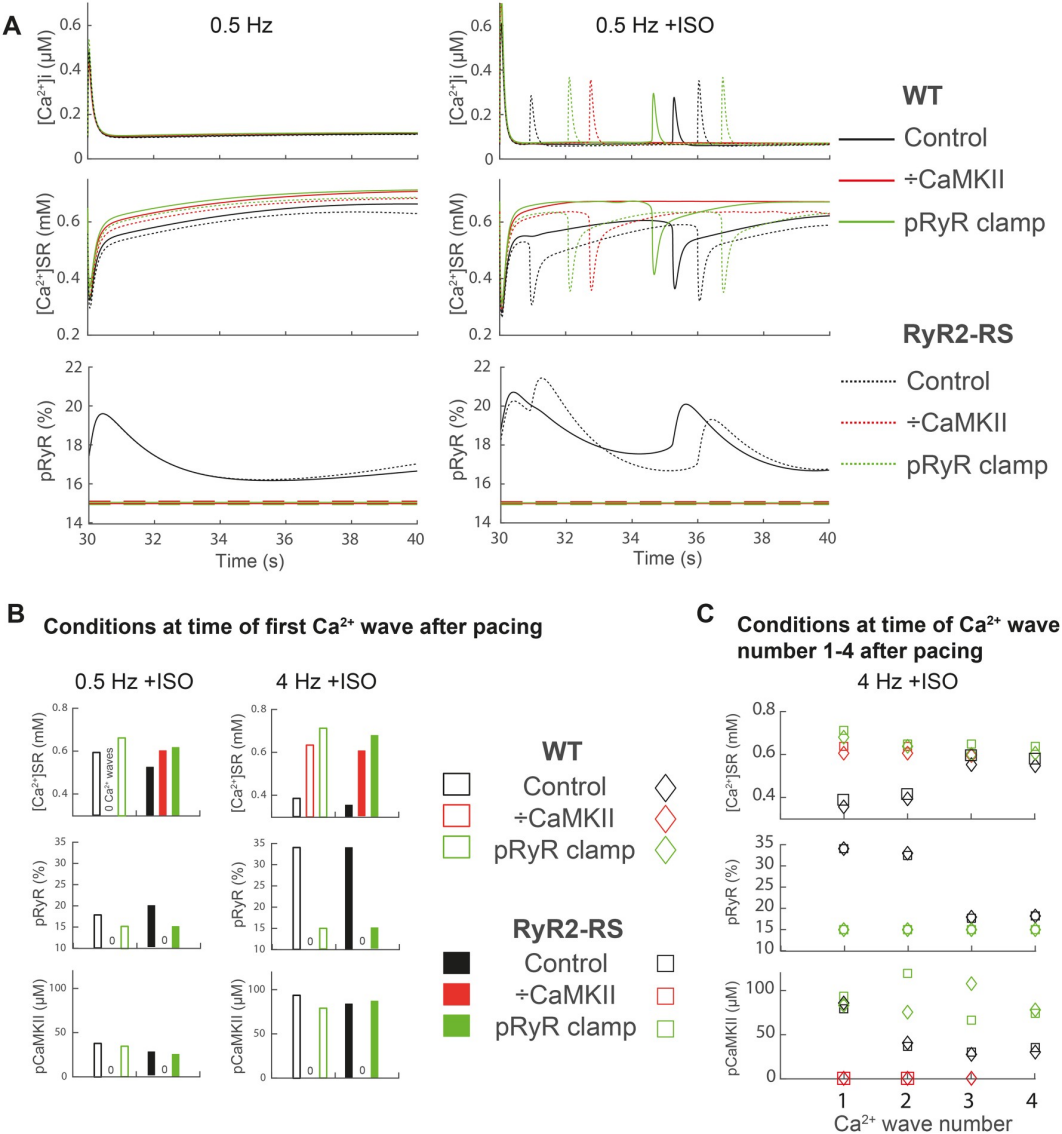
Computational modelling elucidated the interaction between pacing frequency and βAR stimulation in Ca^2+^ wave development. (A) Modelled representation of whole-cell intracellular Ca^2+^ ([Ca^2+^]_i_) and SR Ca^2+^ content ([Ca^2+^]_SR_), as well as fluctuations in the level of RyR2 phosphorylation (pRyR) relative to a quiescent myocyte, in a 10 s period after pacing at 0.5 Hz in absence (left panel) and presence of ISO (right panel). Similar simulations were performed with 4 Hz pacing (not shown). The impact of CaMKII and phosphorylation of RyR2 was tested by omitting CaMKII from the model entirely (÷CaMKII), and by holding RyR2 phosphorylation to a set level found in quiescent cells in absence of ISO (RyR P-clamp). (B) SR Ca^2+^ content ([Ca^2+^]_SR_), level of RyR2 phosphorylation (pRyR), and abundance of autophosphorylated CaMKII (pCaMKII) at the time of occurrence of the first Ca^2+^ wave after 0.5 and 4 Hz pacing in absence and presence of ISO. Note that after 0.5 Hz in absence of ISO no Ca^2+^ waves occurred in WT. (C) SR Ca^2+^ content, level of RyR2 phosphorylation (RyR-P), and CaMKII activity at the time of the first and subsequent Ca^2+^ waves in a 10 s period after 4 Hz pacing.

A computational model of mouse ventricular cardiomyocyte electrophysiology and ion homeostasis was employed to deconvolve the factors underlying the effects of pacing frequency and βAR stimulation on Ca^2+^ wave propensity.[24] With a 10% increase in RyR2 luminal Ca^2+^ sensitivity, the model reproduced the effects of pacing and βAR stimulation on Ca^2+^ wave frequency and latency in a 10 s post pacing period (S2 Fig). The model allowed a continuous readout of intracellular Ca^2+^, SR Ca^2+^ content, CaMKII activity, and level of CaMKII-dependent phosphorylation of RyR2 at serine 2814. The following observations were made regarding the development of Ca^2+^ waves in the post-pacing period (Fig 6): First, at 0.5 Hz βAR stimulation was necessary for Ca^2+^ waves to occur in both RyR2-RS and WT (Fig 6A, left vs. right upper panels). Second, when all effects of CaMKII was removed or RyR2 phosphorylation level was kept at baseline, i.e. without effects of pacing or βAR stimulation, Ca^2+^ wave propensity decreased in both RyR2-RS and WT (Fig 6A, red and green lines). Third, higher pacing frequency increased the propensity for Ca^2+^ waves by increased activation of CaMKII and phosphorylation of RyR2 (Fig 6B, left vs right panels). However, higher frequency also increased the propensity for Ca^2+^ waves by increasing SR Ca^2+^ content, but only in WT (Fig 6B). Fourth, the main effect of CaMKII on Ca^2+^ wave propensity depended on the time after cessation of pacing: Early after pacing, CaMKII activity and RyR2 phosphorylation were high, allowing Ca^2+^ waves to initiate at a low SR Ca^2+^ content. Ca^2+^ waves occurred earlier in RyR2-RS because the SR Ca^2+^ threshold for Ca^2+^ waves was intrinsically lower than in WT, i.e. the SR Ca^2+^ content at the time of the first Ca^2+^ wave was lower in RyR2-RS (6A-C). Later in the diastolic period, the degree of RyR2 phosphorylation decayed more rapidly than the SR Ca^2+^ refilling, and further Ca^2+^ waves could only be generated by increased SR Ca^2+^ content caused by reduced CaMKII-dependent RyR2 phosphorylation (Fig 6C), and subsequent reduced SR Ca^2+^ leak. With removal of CaMKII-dependent effects on Ca^2+^ handling or by keeping RyR2 phosphorylation level at the baseline for the model, the threshold SR Ca^2+^ content for waves was high even in the early phase of diastole, and time for refilling dominated the Ca^2+^ wave latency (Fig 6A and 6C).

## 4. Discussion

Our study confirms that ventricular arrhythmias in patients with CPVT1 are associated with increasing heart rate during exercise testing, as previously reported.[1, 21] However, comparisons of bicycle testing to ICD pacing indicate that increased heart rate by itself is not sufficient to induce arrhythmias. Experiments with ventricular cardiomyocytes corroborated this conclusion: while high pacing frequencies increased the number of Ca^2+^ waves in both RyR2-RS and WT, βAR stimulation was necessary to reveal the increased propensity for Ca^2+^ waves associated with CPVT1 in RyR2-RS. Computer simulations of CPVT1 cardiomyocytes further strengthened these findings, and show that although higher pacing frequency promotes Ca^2+^ wave development, the effects of βAR stimulation on SR Ca^2+^ release dynamics are necessary to allow increased propensity for Ca^2+^ waves during high pacing frequencies in RyR-RS cardiomyocytes.

Previous studies have established that βAR stimulation increases the degree of SR Ca^2+^ leak and the propensity for arrhythmias in CPVT1,[26] while the effect of pacing frequency *per se* has not yet been studied in this condition. Based on previous studies, we had reason to believe that frequency could promote SR Ca^2+^ leak, and that this could be partly CaMKII-dependent: In ventricular cardiomyocytes, both high heart rate and βAR stimulation increases the activity of CaMKII, which has been shown to increase SR Ca^2+^ leak.[18, 19] The mechanism for activation of CaMKII during increased pacing frequencies is high cytosolic Ca^2+^ concentration.[27] The importance of CaMKII-dependent SR Ca^2+^ leak in CPVT1 is further indicated by the fact that CaMKII-inhibition by KN-93 or autocamtide-1 related inhibitory peptide reduced spontaneous Ca^2+^ release in ventricular cardiomyocytes from mice with the CPVT1-causative *RyR2*-R4496C mutation.[19] Our results support an important role for CaMKII in RyR2-RS, although the mechanism is somewhat more complex than previously hypothesized.

SR Ca^2+^ leak is highly dependent on SR Ca^2+^ content. In our study, SR Ca^2+^ content did not change with increasing pacing frequency or βAR stimulation in RyR2-RS cardiomyocytes. However, compared to WT, Ca^2+^ transient amplitude in RyR2-RS went from equal or higher without βAR stimulation, to lower during βAR stimulation. This is in contrast to findings in *RyR2*-R4496C cardiomyocytes in which Ca^2+^ transient amplitude was not changed by βAR stimulation.[19] One explanation for our findings is that the RyR2 is slightly sensitized even in absence of βAR stimulation, resulting in an increased fractional release even at baseline. Indeed, SR Ca^2+^ threshold for Ca^2+^ waves in RyR2-RS was lower even in absence of βAR stimulation. Thus, one interpretation of our results is that when βAR stimulation is added, SR Ca^2+^ leak increases more than Ca^2+^ homeostatic mechanisms can compensate for, resulting in decreased SR Ca^2+^ content and Ca^2+^ transient amplitude.

We used the computational model to further elucidate the effects of βAR stimulation: The model shows that in absence of βAR stimulation, the rate of SR Ca^2+^ refilling is insufficient to increase the propensity for Ca^2+^ waves. However, in presence of βAR stimulation, SR Ca^2+^ refilling in combination with a decreased threshold for SR Ca^2+^ release is sufficiently fast for early initiation of Ca^2+^ waves. These results are in line with conclusions from the *RyR2-*R4496C CPVT1 mouse model,[26].

Still, two possible mechanisms for the increased number of Ca^2+^ waves seen during βAR stimulation: either that βAR stimulation is necessary to increase SR Ca^2+^ content sufficiently for release, or that such stimulation further destabilizes RyR2 and thereby decreases the SR Ca^2+^ threshold for Ca^2+^ release. Our experimental results did not show a change in this threshold in RyR2-RS cardiomyocytes during βAR stimulation compared to baseline. This could indicate that the main effect of βAR stimulation is to increase SR Ca^2+^ content sufficiently for release. Interestingly, this interpretation is partly supported by our computational model: The major observation across simulations was that the propensity for Ca^2+^ waves to occur depended most critically on two factors that vary in time during the diastolic interval. The first factor is the degree of CaMKII phosphorylation at RyR2. This is because, in this model, increasing CaMKII-dependent phosphorylation of RyR2 reduces the threshold SR Ca^2+^ load for a Ca^2+^ wave. The degree of phosphorylation at any time after a beat depends on both the peak level achieved during pacing, and the rate of dephosphorylation in the period after the beat. The second factor is the rate at which SR Ca^2+^ load is restored during the diastolic interval. Because RyR2 dephosphorylation dynamically increases the threshold SR Ca^2+^ content after each beat, while the SR is simultaneously refilling with Ca^2+^, it is the combination of these two dynamic effects that determines when a Ca^2+^ wave will occur in this model. βAR stimulation promotes Ca^2+^ waves because it both increases RyR phosphorylation by CaMKII, and dramatically increases the rate of SR Ca^2+^ refilling. Increased pacing frequency also exaggerates both of these effects, but more modestly.

A potentially important observation from our computational model with regard to diastolic Ca^2+^ release is that the effects of CaMKII may be highly dynamic in cardiac myocytes, even during an individual cardiac contraction-relaxation cycle, which might explain why an increase in CaMKII-dependent phosphorylation of RyR2-Ser2814 during ISO stimulation was not detected by immunoblotting. However, changes in RyR2-Ser2814 phosphorylation have been well documented in chronic disease models by previous studies.[18] While the relevance of the observations made from pause-induced release experiments (and simulations) to clinical exercise stress tests is limited, the finding that RyR2 phosphorylation is important in early diastole may help to explain why blockade of CaMKII alleviates the pro-arrhythmia associated with CPVT1-causative mutations.[19]

The discussion above illustrates the complexity of Ca^2+^ homeostasis in ventricular cardiomyocytes at the core of arrhythmia development in CPVT1. However, the complete understanding of arrhythmia development even in this monogenic disease requires even further levels of complexity as both βAR stimulation and heart rate also affects the propensity for DADs to trigger action potentials and the propensity for development of VT.[16, 28] These aspects are beyond the scope of our study. Also important are electrophysiological differences between the human and mouse heart for interpretation of our data: In humans, SR Ca^2+^ content increases with increasing pacing frequencies, contributing to a positive force-frequency relationship; in contrast, mice exhibit a less steep or no increase in SR Ca^2+^ content during increased pacing frequencies.[29] Furthermore, due to a 10-fold faster heart rate and shorter action potentials the mouse is different from the human heart. To capture spontaneous Ca^2+^ release events occurring during diastole in isolated myocytes, experiments were performed at artificially slow pacing frequencies compared to in vivo heart rates for mice. Because relatively slow pacing can affect the probability of these events, further validation in human cardiomyocytes and in vivo models is warranted.

In our study, direct observations of heart rate effects in patients were made in the ICD-based pacing procedure, which indicated that increased heart rate *per se* was not sufficient to induce arrhythmias. However, the pacing protocol have important limitations: F.ex., during the pacing protocol, heart rate was increased for a shorter time than during the bicycle exercise test. We can only speculate that this could affect CaMKII activation. Our results show that the untangling of the effects of cathecolamines and heart rate should be further pursued in future studies, including extended and comprehensive pacing procedures.

In conclusion, our clinical and experimental data, as well as computational modelling, show that increased heart rate and heart rate-independent effects of βAR stimulation on ventricular cardiomyocytes combine to increase the risk of arrhythmias, with βAR stimulation being the necessary factor to reveal the CPVT1 phenotype in *RyR2*-R2474S. These data supports that the mainstay of treatment for CPVT1 is antagonism of βAR stimulation in ventricular cardiomyocytes.

## Supporting information

S1 Fig Western blots. Complete western blots with molecular size markers used in Fig 5. Blue arrows indicate analysed band. (A) GAPDH (goat polyclonal antibody), (B) CaMKII (rabbit polyclonal antibody), (C) pCaMKII (rabbit polyclonal antibody), (D) RyR (mouse monoclonal antibody), (E) pRyR Ser2808 (rabbit polyclonal antibody), (F) pRyR Ser2814 (rabbit polyclonal antibody), (G) PLB (mouse monoclonal antibody), (H) pPLB Ser16 (rabbit polyclonal antibody), (I) pPLB Thr17 (rabbit polyclonal antibody) and (J) SERCA2a (mouse monoclonal antibody).
(PDF)

S2 Fig Computational model. The effects of increased pacing frequency and stimulation of βAR on RyR-RS and WT observed in cellular experiments were reproduced in a computational model of mouse ventricular myocyte electrophysiology and ion homeostasis. The only adaptation needed to recapitulate the difference between RyR-RS and WT was increased luminal RyR2 Ca2+ sensitivity by 10% in RyR-RS. (A) The left panel shows modelled representation of whole-cell intracellular Ca^2+^ ([Ca^2+^]i) and SR Ca^2+^ content ([Ca^2+^]SR) during the last three seconds of a pacing protocol similar to the one used in the cellular experiments, while the right panel shows the same parameters in a 10 s post-pacing period after cessation of pacing. (B) Bar graphs of results from the post-pacing period: The left panel shows the frequency of Ca^2+^ waves in a 10 s period after 0.5 and 4 Hz pacing in presence and absence of ISO. Increased pacing frequency increased the frequency of Ca^2+^ waves in the post-pacing period in both RyR2-RS and WT, while ISO increased the Ca^2+^ wave frequency more in RyR2-RS. The right panel shows the time to occurrence of the first Ca^2+^ wave after cessation of pacing, i.e. Ca^2+^ wave latency. Increased pacing frequency decreased Ca^2+^ wave latency in a post-pacing period in both RyR-RS and WT, while ISO decreased the Ca^2+^ wave latency more in RyR2-RS.F.
(PDF)
