# Supplementary material for: Arrhythmia initiation in catecholaminergic polymorphic ventricular tachycardia type 1 depends on both heart rate and sympathetic stimulation
Source: PLoS One. 2018 Nov 6;13(11):e0207100. doi: 10.1371/journal.pone.0207100 (PMC6219810; doi:10.1371/journal.pone.0207100)

A) GAPDH low frequency (4 Hz):

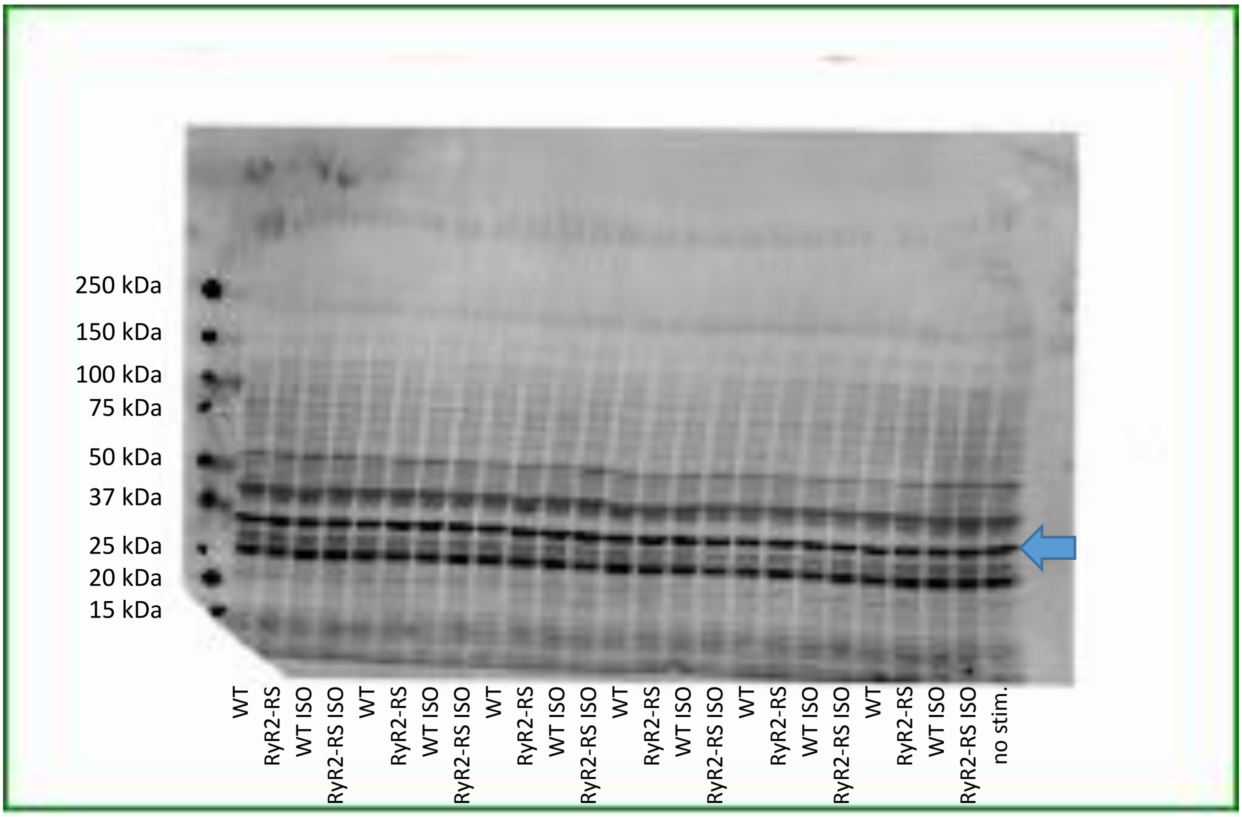

GAPDH high frequency (8 Hz):

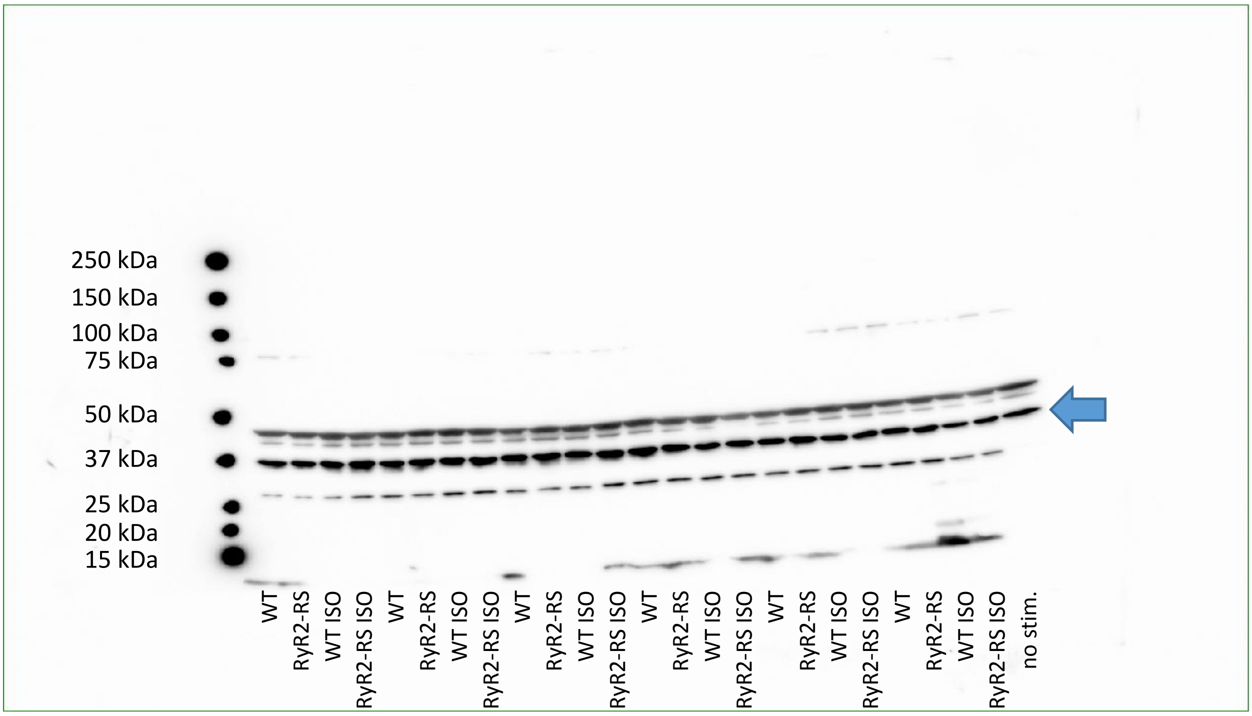

B) **CaMKII low frequency (4 Hz):**

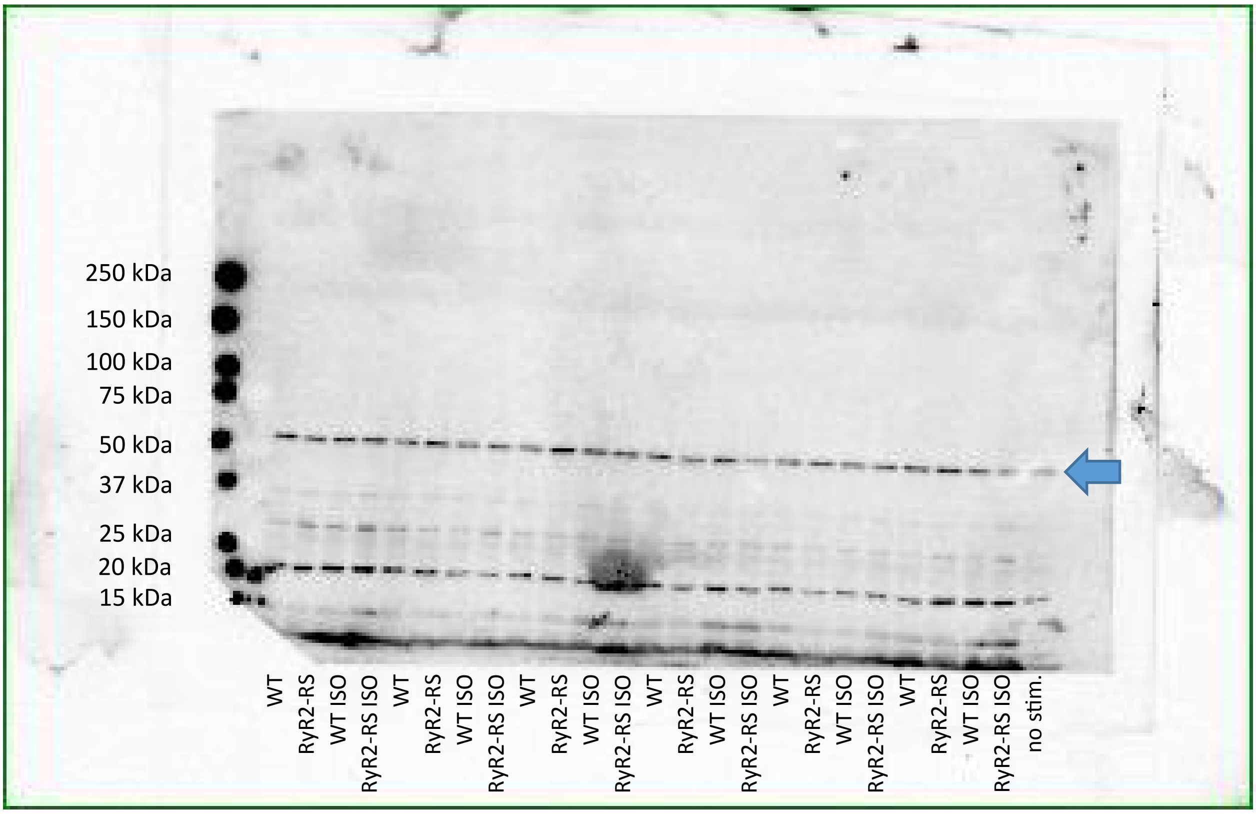

CaMKII high frequency (8 Hz):

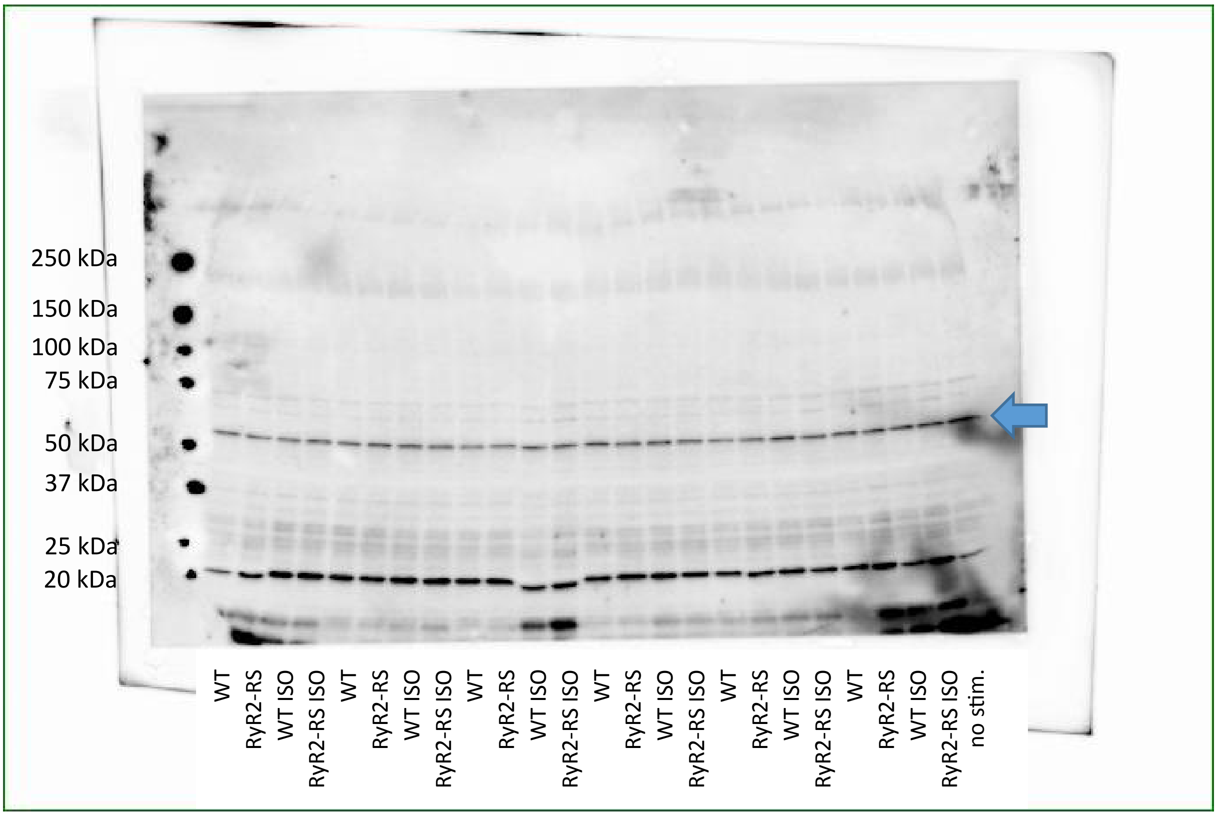

C) **pCaMKII low frequency (4 Hz):**

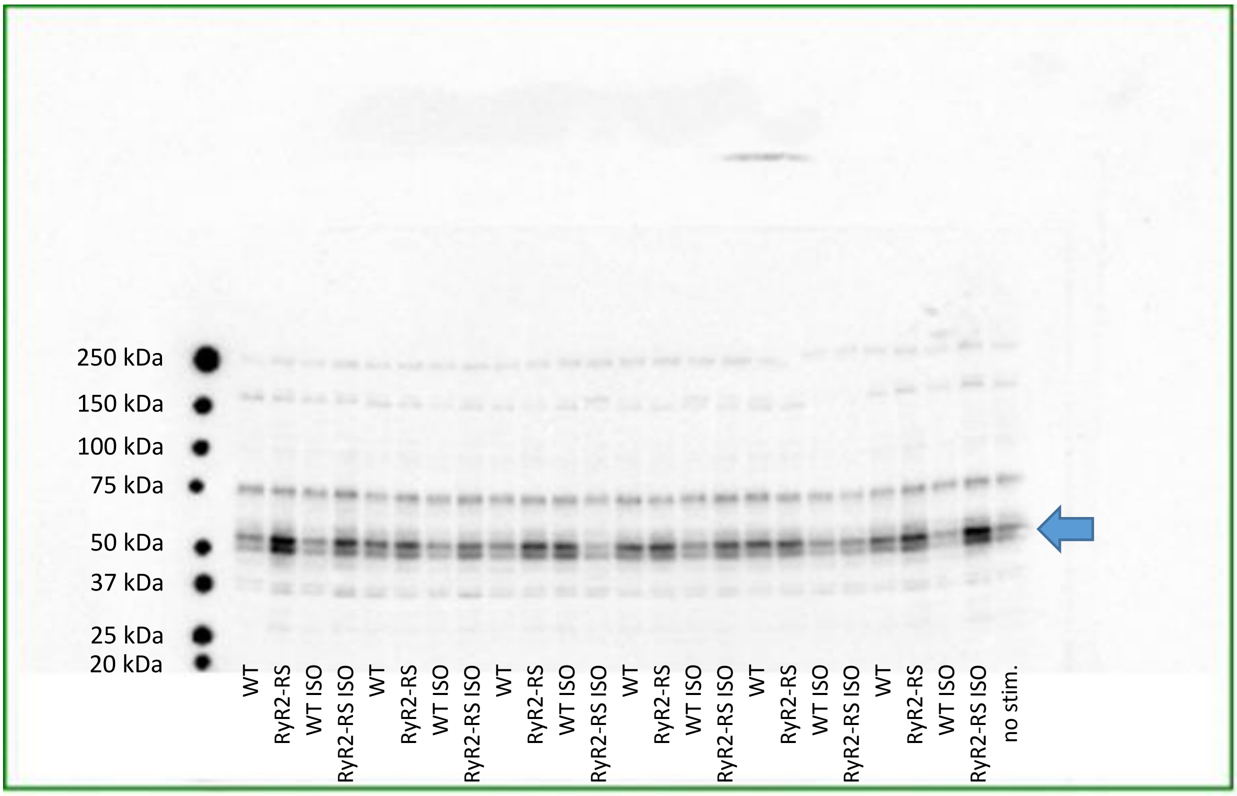

pCaMKII high frequency (8 Hz):

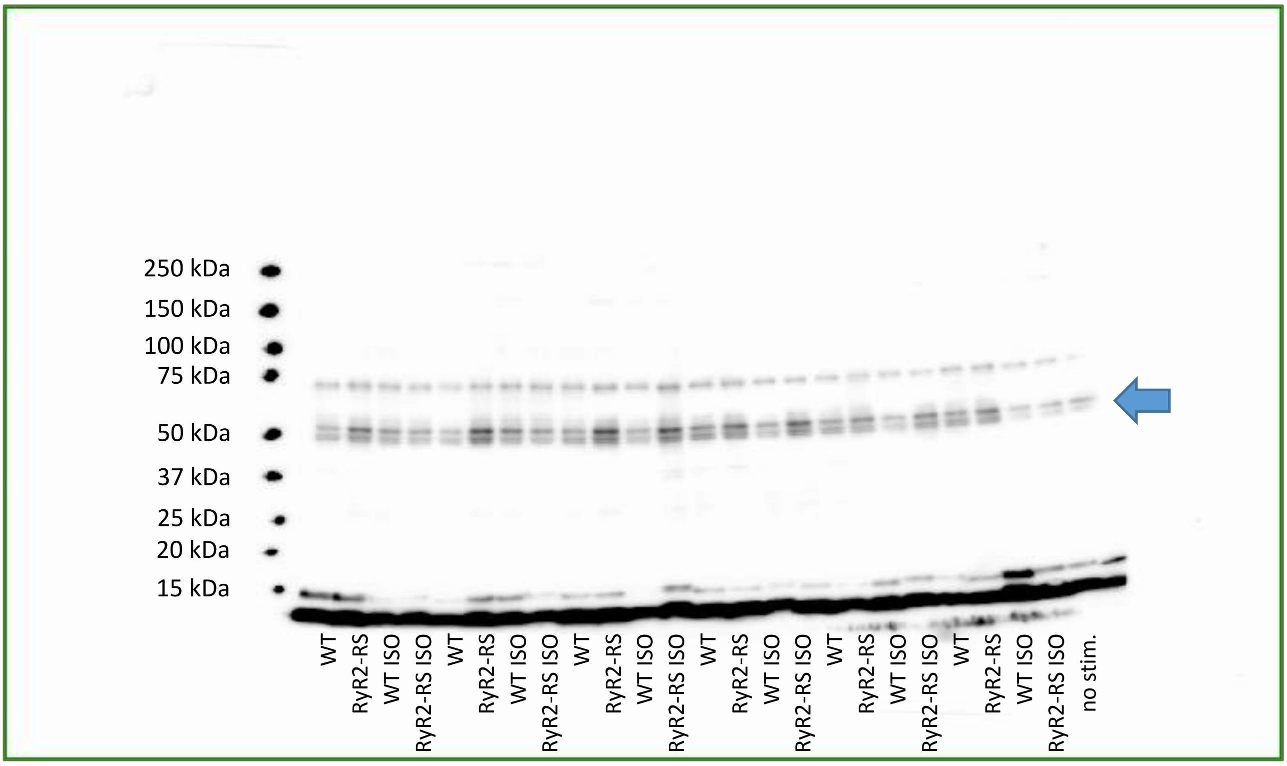

D) RyR low frequency (4 Hz):

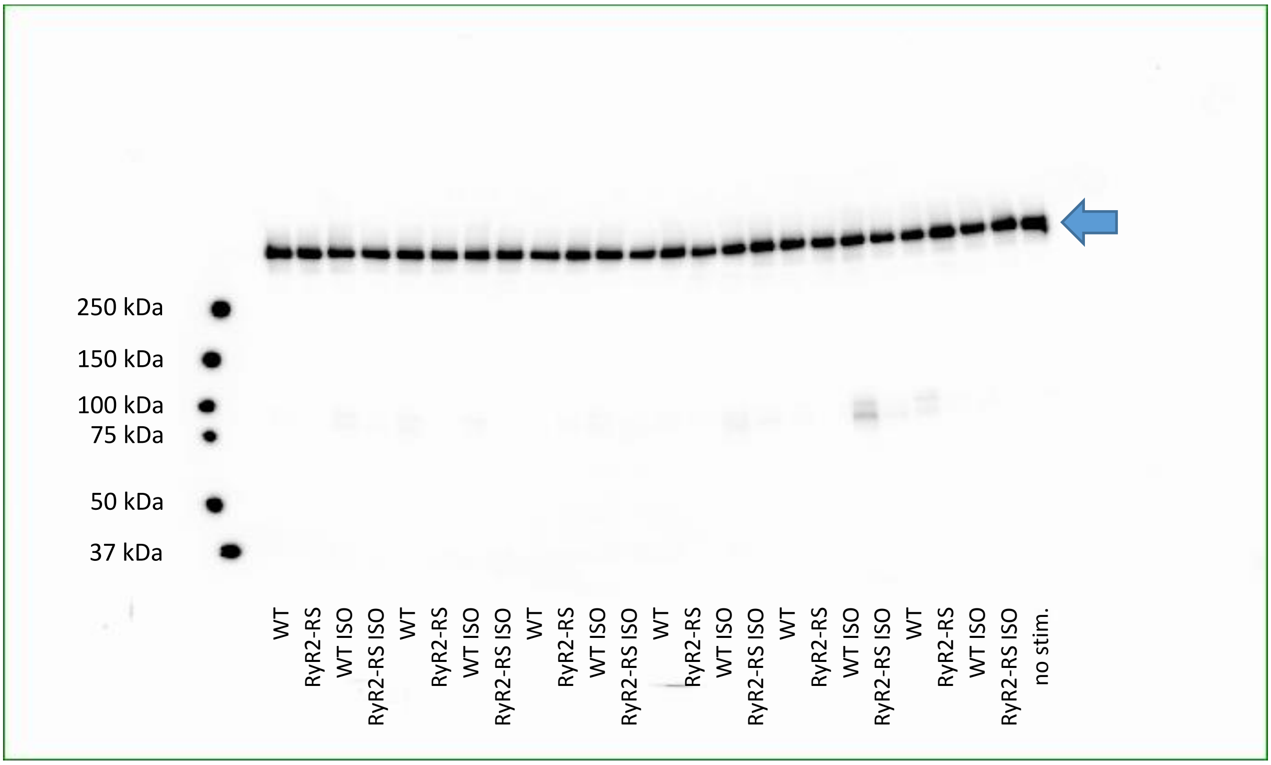

RyR high frequency (8 Hz):

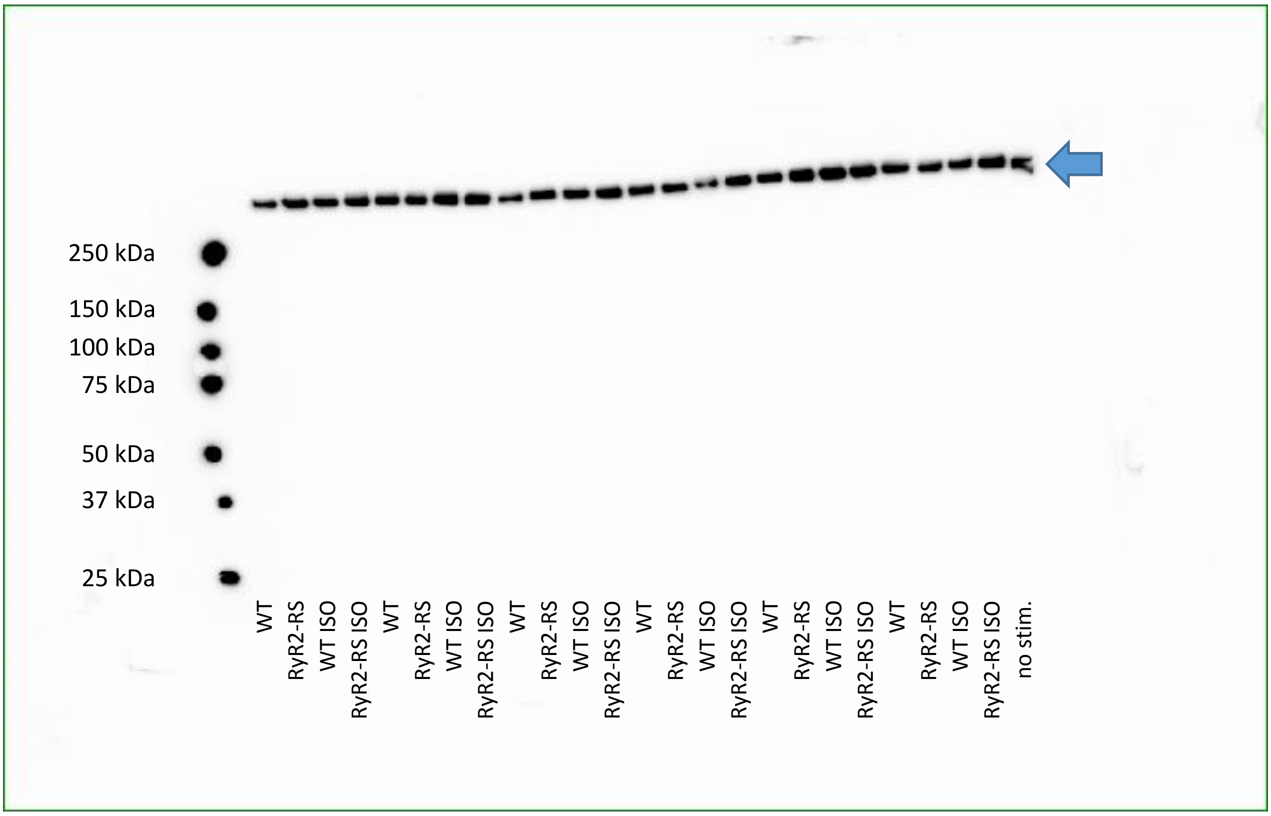

E) pRyR 2808 low frequency (4 Hz):

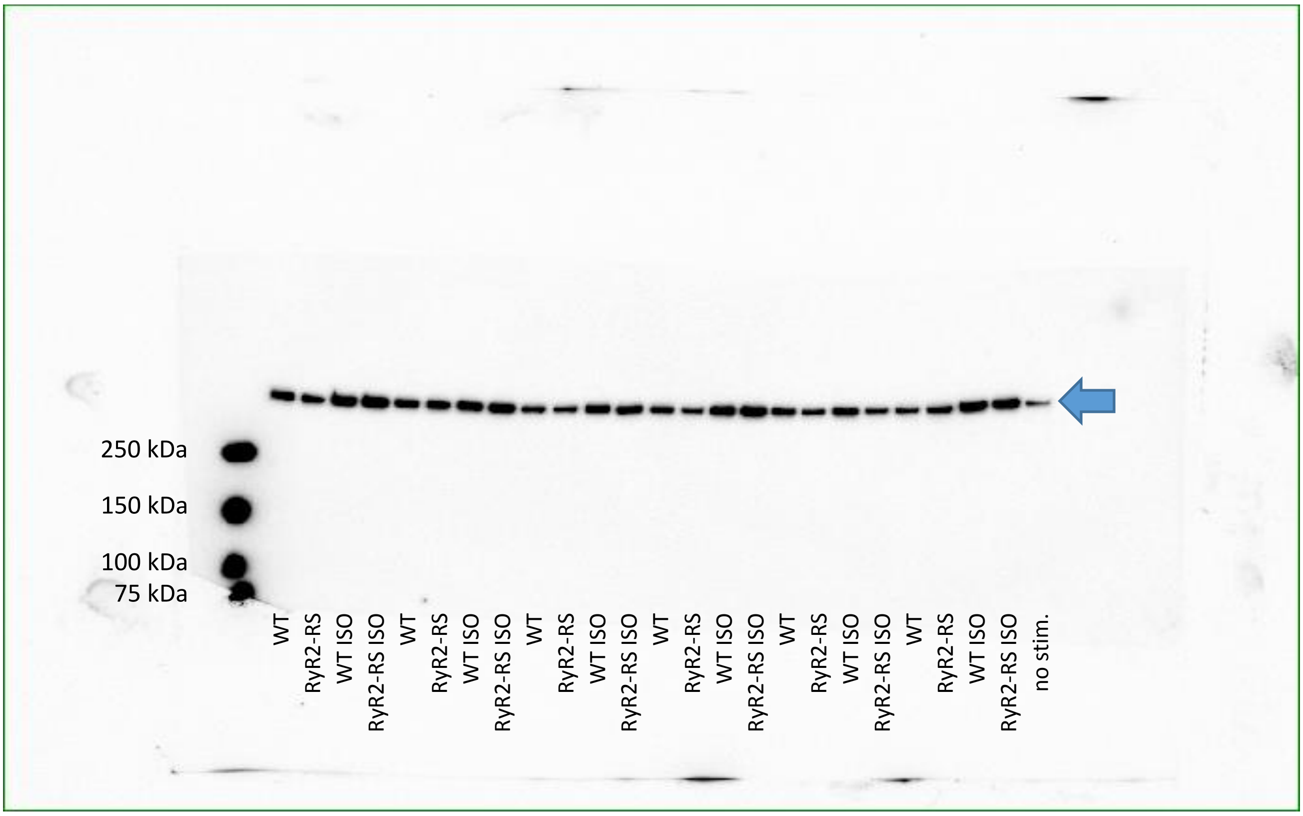

pRyR 2808 high frequency (8 Hz):

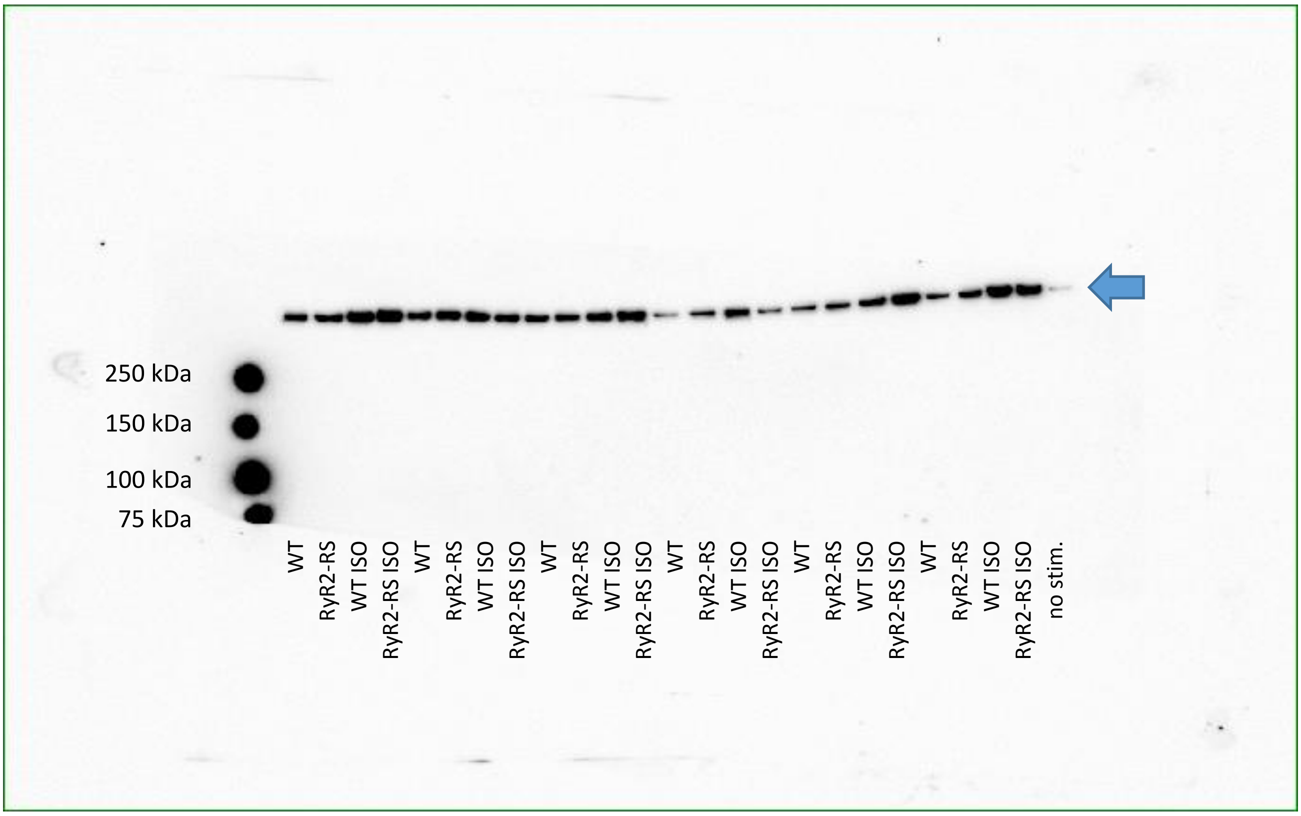

F) pRyR 2814 low frequency (4 Hz):

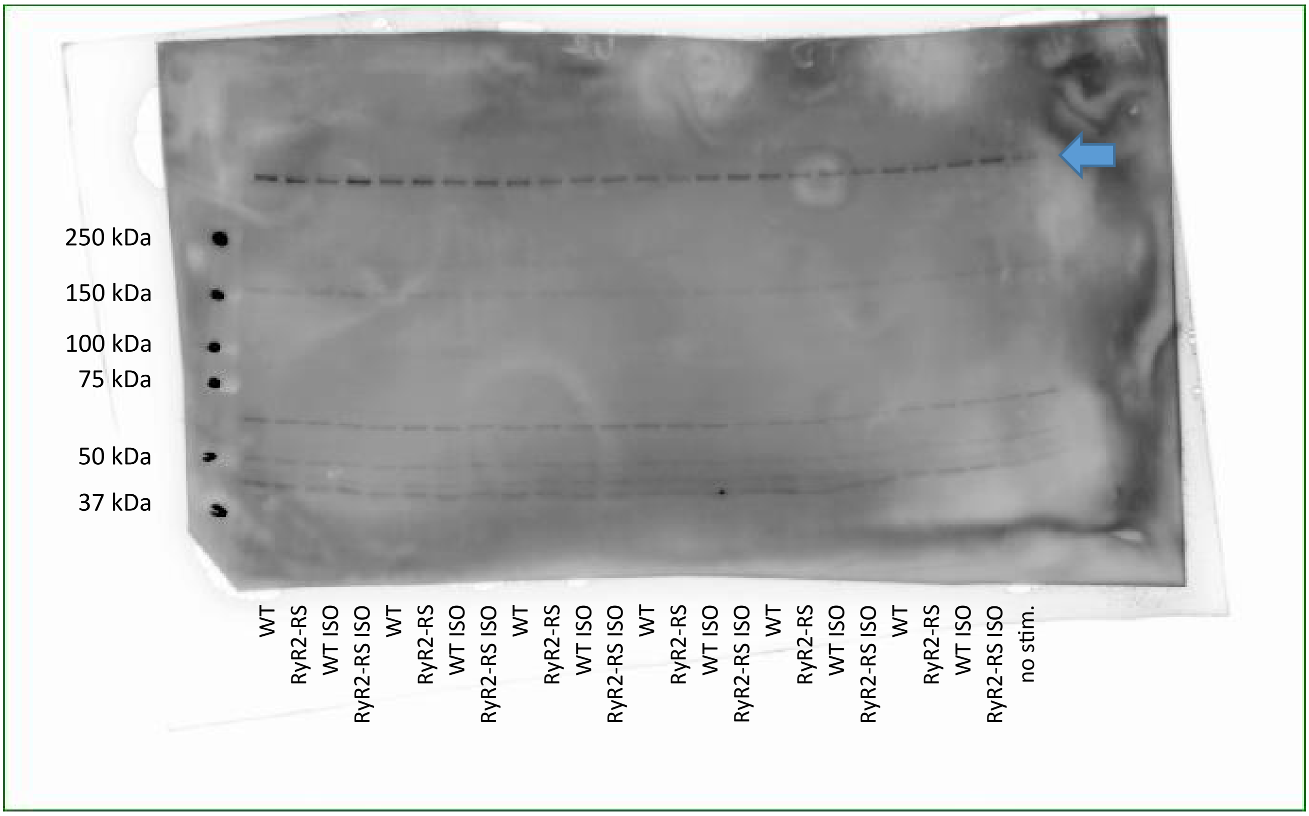

pRyR 2814 high frequency (8 Hz):

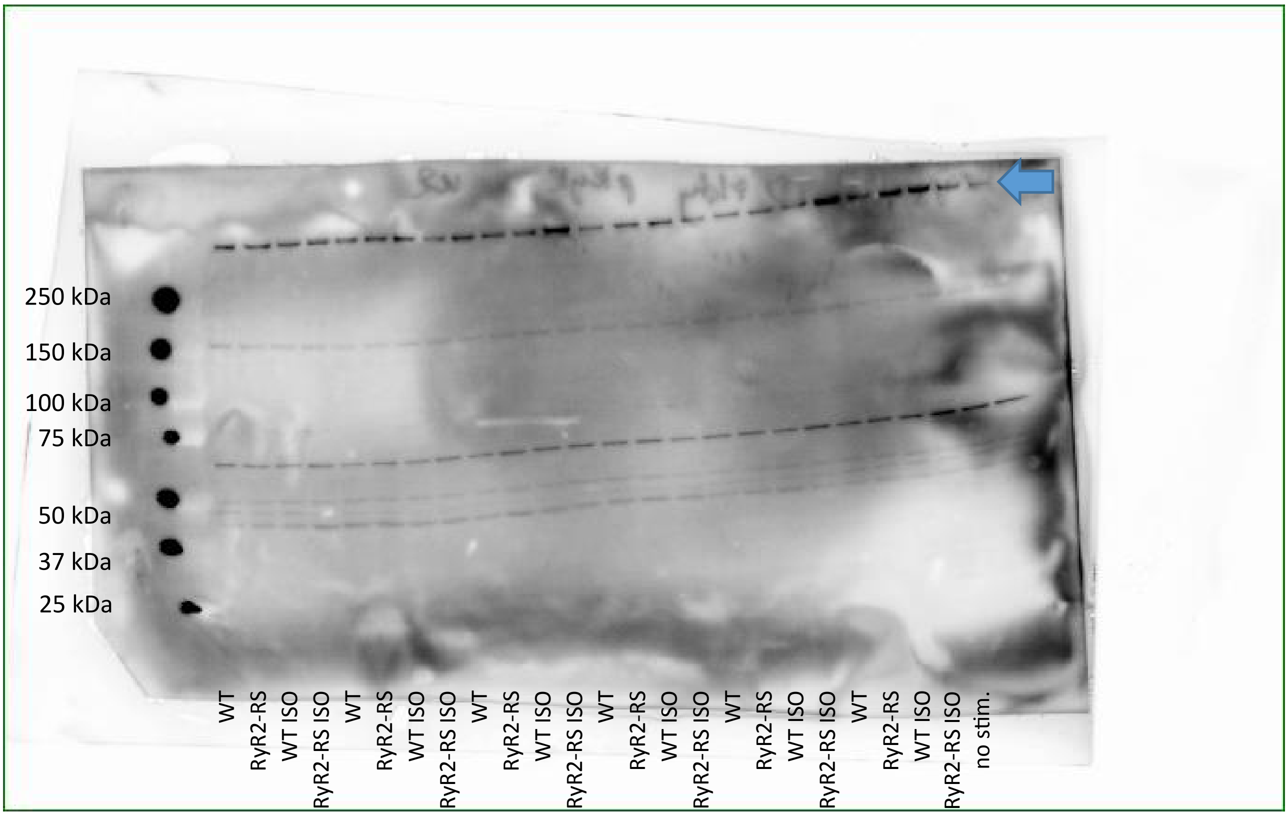

G) PLB low frequency (4 Hz):

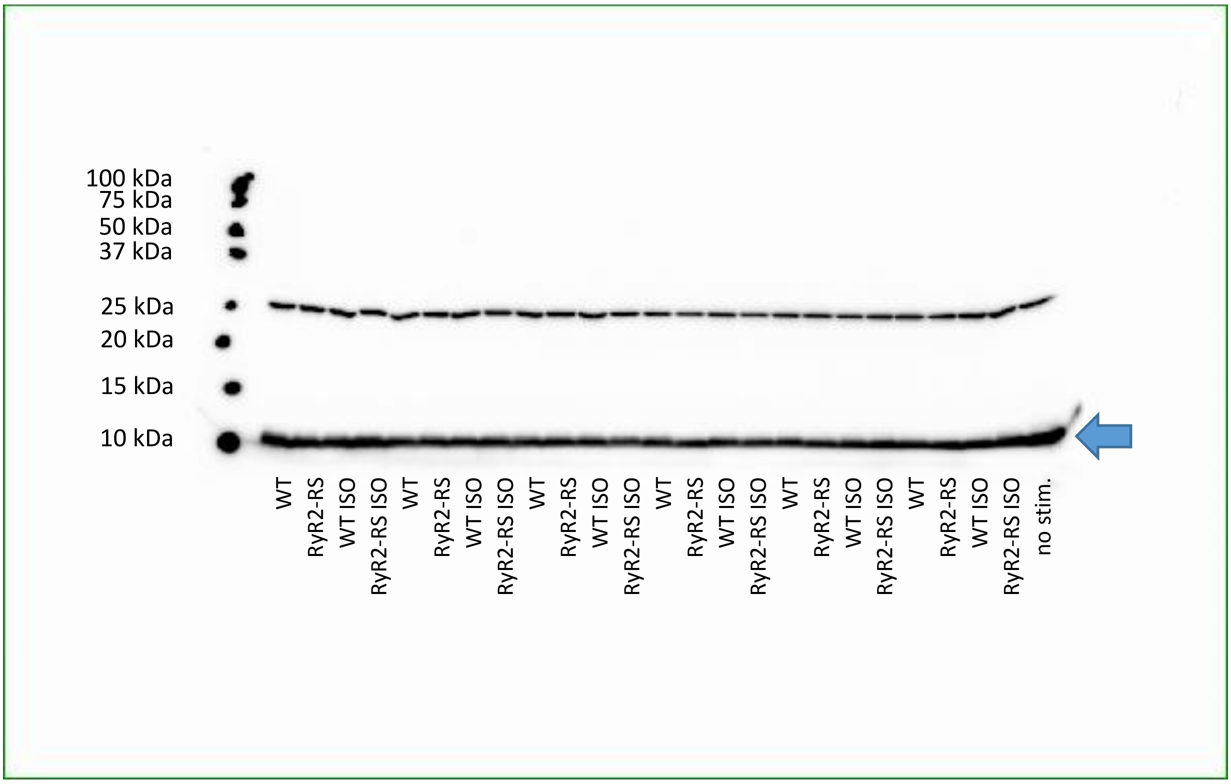

PLB high frequency (8 Hz):

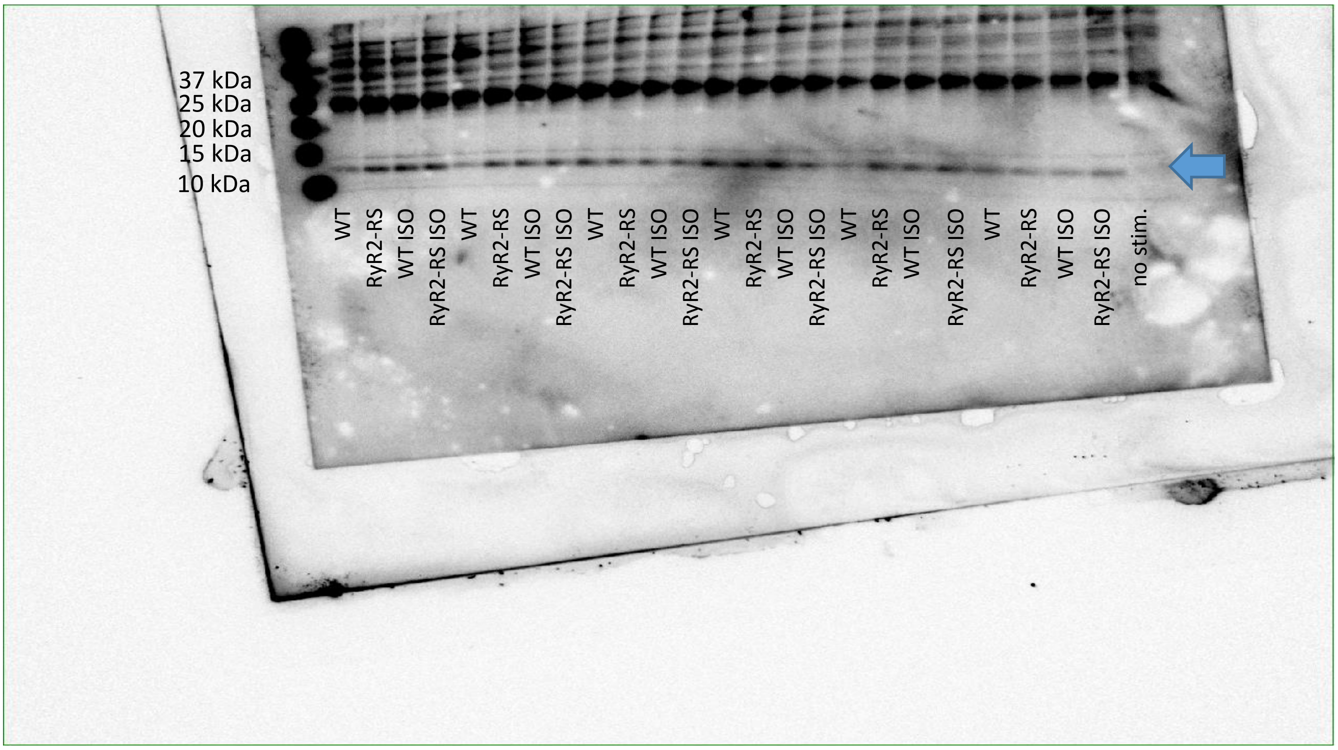

H) pPLB Ser16 low frequency (4 Hz):

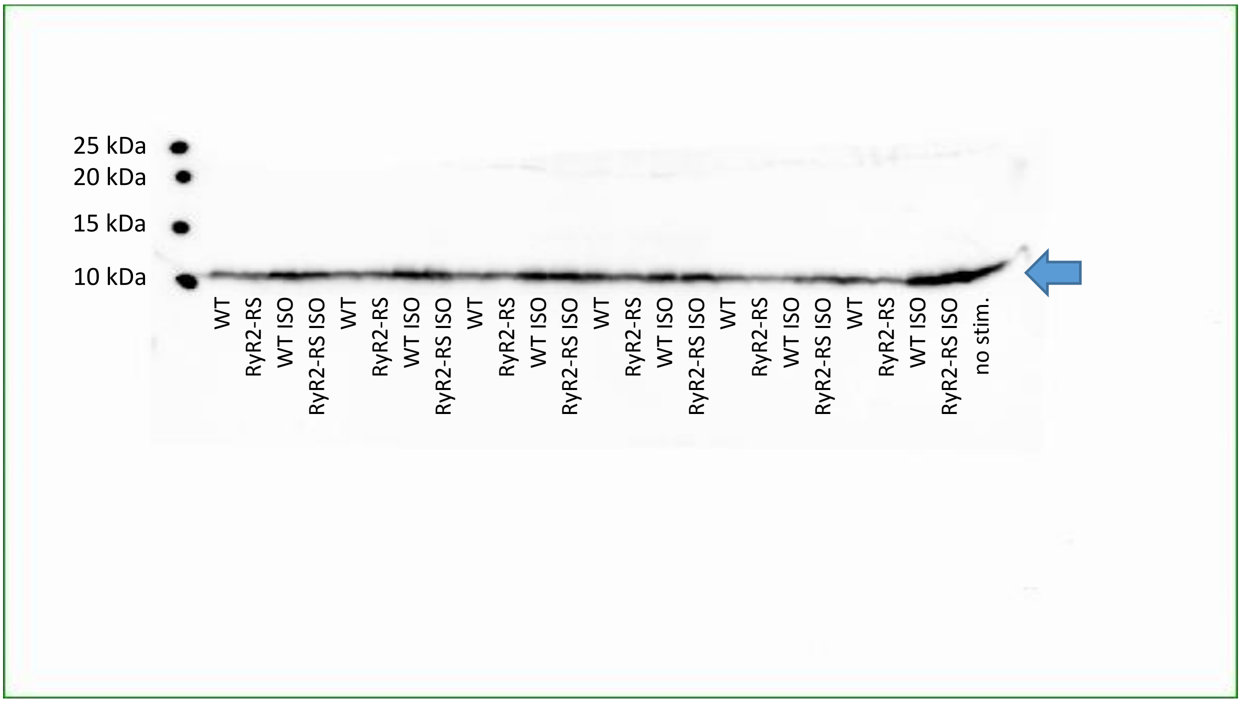

pPLB Ser16 high frequency (8 Hz):

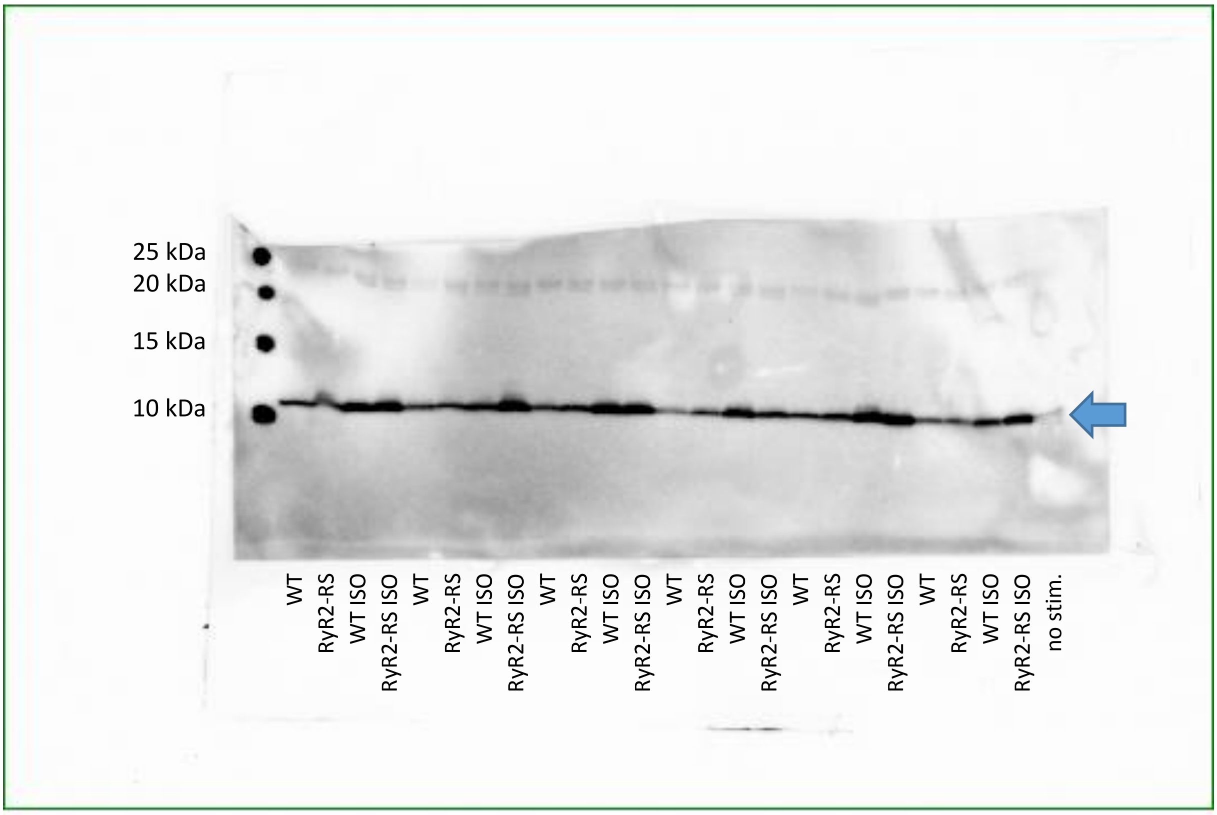

I) **pPLB Thr17 low frequency (4 Hz):**

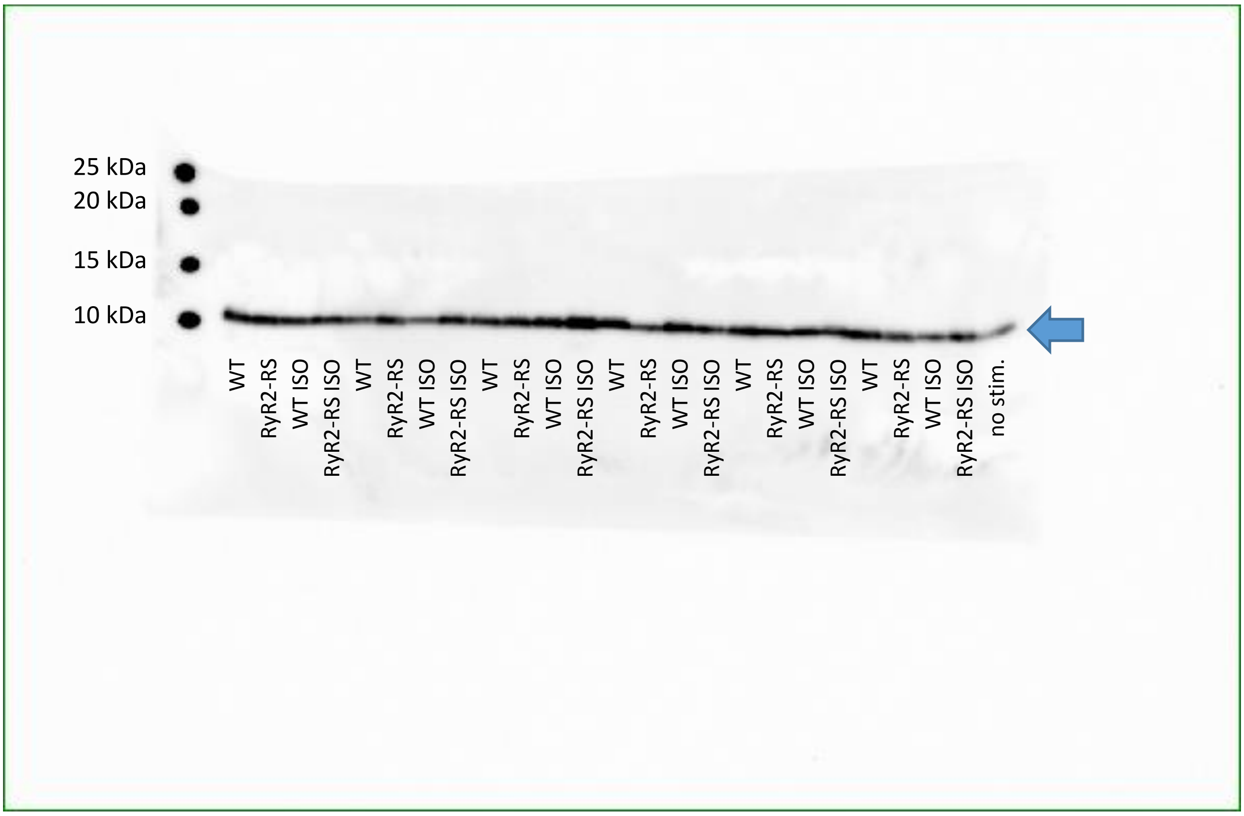

pPLB Thr17 high frequency (8 Hz):

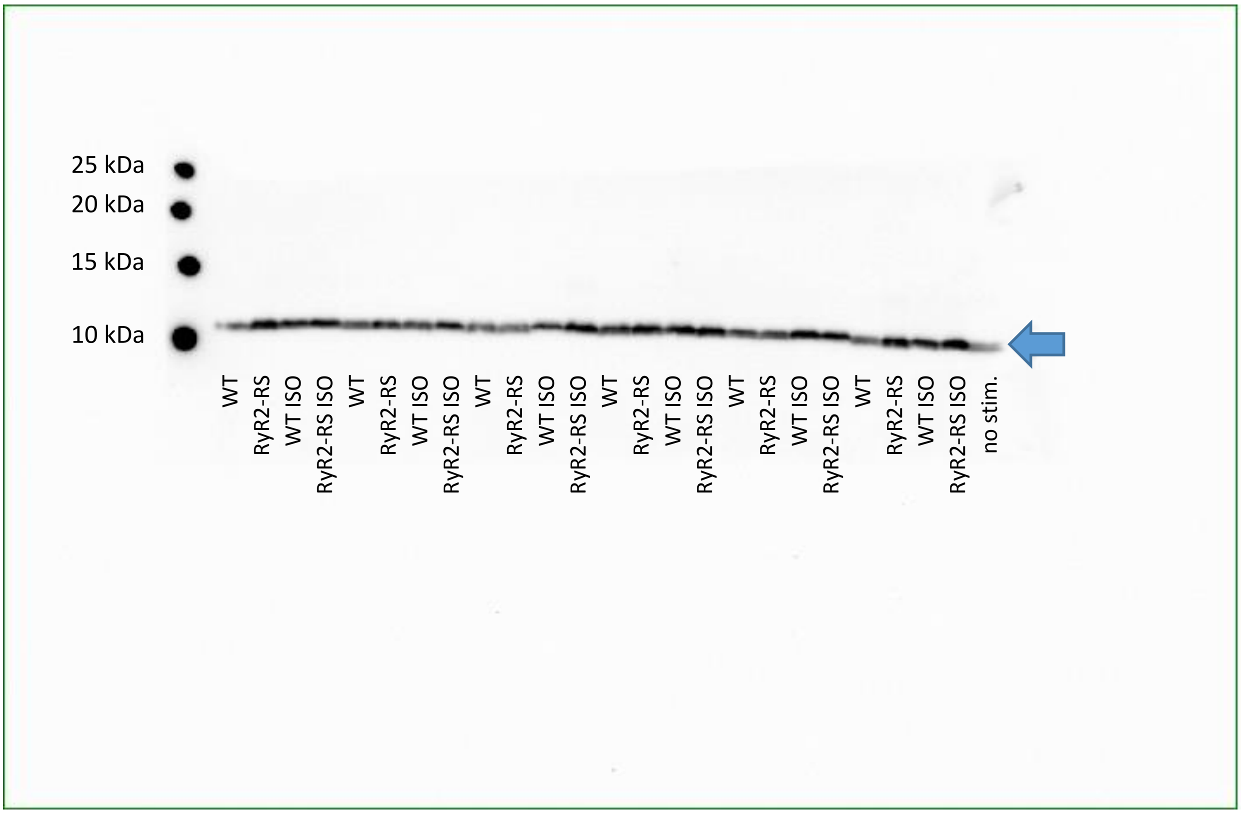

J) SERCA2a low frequency (4 Hz):

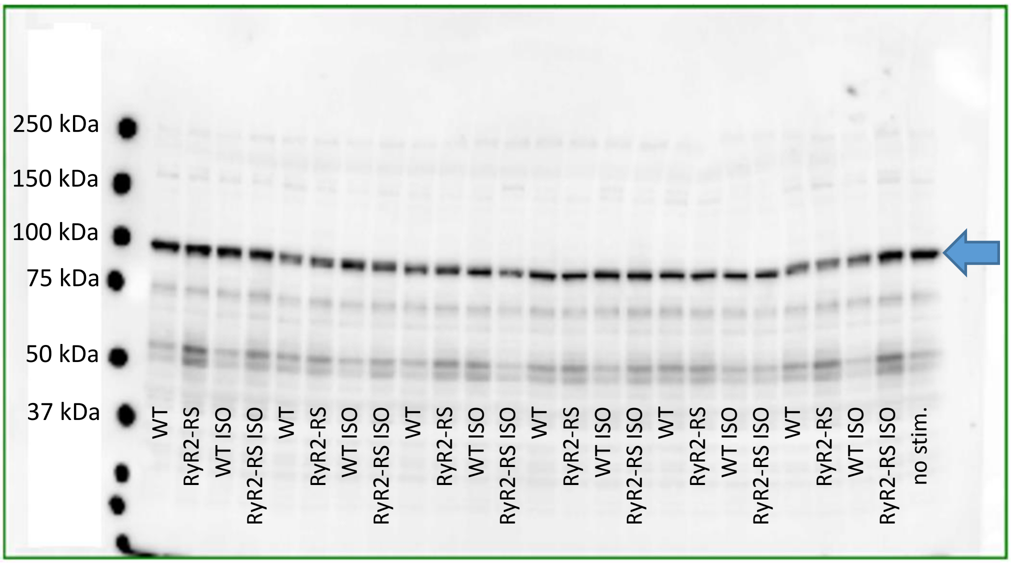

SERCA2a high frequency (8 Hz):

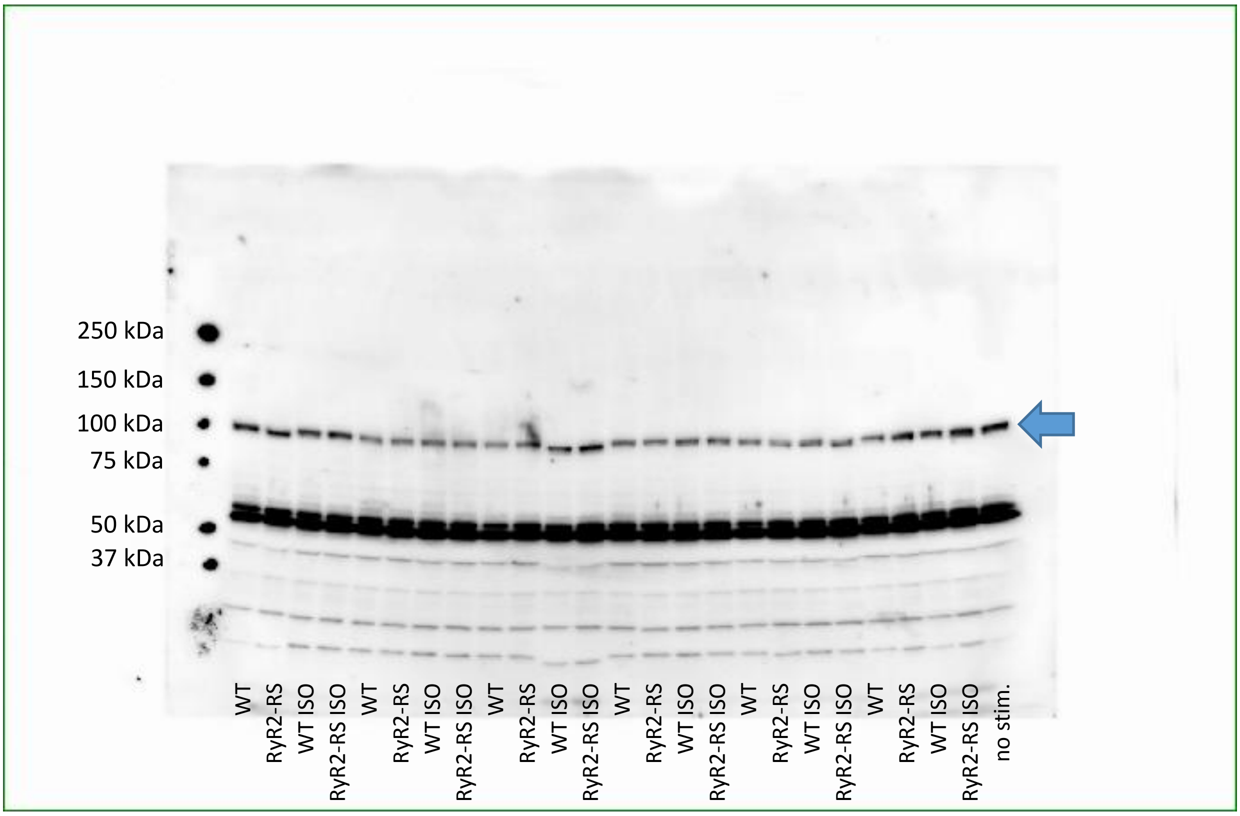

Supplement: S1 Fig — Complete western blots with molecular size markers used in Fig 5. Blue arrows indicate analysed band. (A) GAPDH (goat polyclonal antibody), (B) CaMKII (rabbit polyclonal antibody), (C) pCaMKII (rabbit polyclonal antibody), (D) RyR (mouse monoclonal antibody), (E) pRyR Ser2808 (rabbit polyclonal antibody), (F) pRyR Ser2814 (rabbit polyclonal antibody), (G) PLB (mouse monoclonal antibody), (H) pPLB Ser16 (rabbit polyclonal antibody), (I) pPLB Thr17 (rabbit polyclonal antibody) and (J) SERCA2a (mouse monoclonal antibody). (PDF) [file pone.0207100.s001.pdf]
