# Supplementary figures and images for: Arrhythmia initiation in catecholaminergic polymorphic ventricular tachycardia type 1 depends on both heart rate and sympathetic stimulation
Source: PLoS One. 2018 Nov 6;13(11):e0207100. doi: 10.1371/journal.pone.0207100 (PMC6219810; doi:10.1371/journal.pone.0207100)

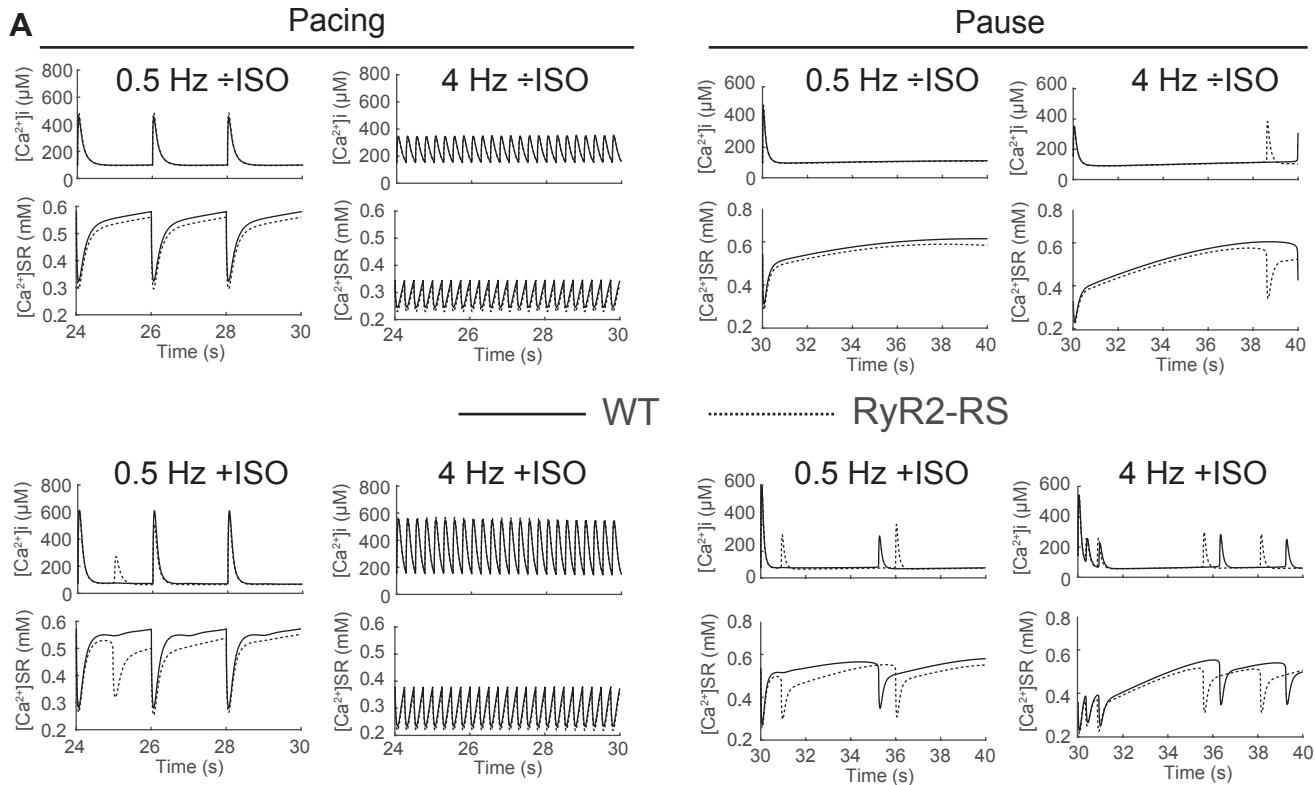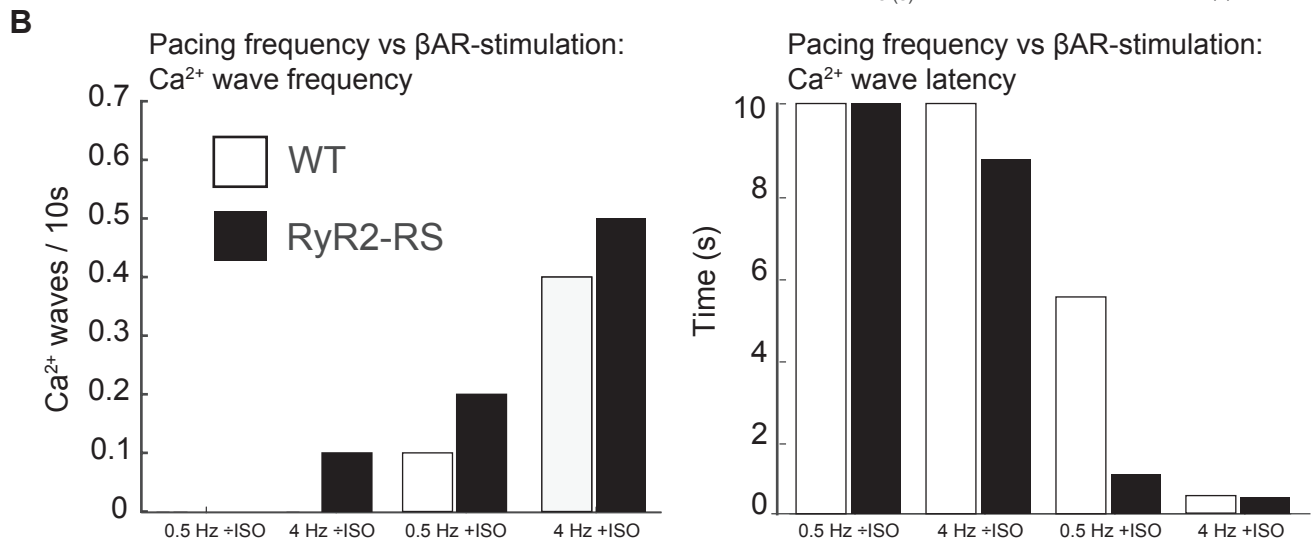

Supplement: S2 Fig — The effects of increased pacing frequency and stimulation of βAR on RyR-RS and WT observed in cellular experiments were reproduced in a computational model of mouse ventricular myocyte electrophysiology and ion homeostasis. The only adaptation needed to recapitulate the difference between RyR-RS and WT was increased luminal RyR2 Ca2+ sensitivity by 10% in RyR-RS. (A) The left panel shows modelled representation of whole-cell intracellular Ca2+ ([Ca2+]i) and SR Ca2+ content ([Ca2+]SR) during the last three seconds of a pacing protocol similar to the one used in the cellular experiments, while the right panel shows the same parameters in a 10 s post-pacing period after cessation of pacing. (B) Bar graphs of results from the post-pacing period: The left panel shows the frequency of Ca2+ waves in a 10 s period after 0.5 and 4 Hz pacing in presence and absence of ISO. Increased pacing frequency increased the frequency of Ca2+ waves in the post-pacing period in both RyR2-RS and WT, while ISO increased the Ca2+ wave frequency more in RyR2-RS. The right panel shows the time to occurrence of the first Ca2+ wave after cessation of pacing, i.e. Ca2+ wave latency. Increased pacing frequency decreased Ca2+ wave latency in a post-pacing period in both RyR-RS and WT, while ISO decreased the Ca2+ wave latency more in RyR2-RS.F. (PDF) [file pone.0207100.s002.pdf]
